# Molecular mechanisms of biomolecular condensate formation in *Drosophila melanogaster* siRNA biogenesis

**DOI:** 10.1093/nar/gkaf664

**Published:** 2025-07-19

**Authors:** Clara Hipp, Selina Mussgnug, Purva Choudhary, Hyun-Seo Kang, Sam Asami, Judit Sastre, Carsten Donau, Romy Böttcher, Gerd Gemmecker, Job Boekhoven, Klaus Förstemann, Michael Sattler

**Affiliations:** Technical University of Munich, TUM School of Natural Sciences, Bavarian NMR Center and Department of Bioscience, 85748 Garching, Germany; Helmholtz Munich, Molecular Targets and Therapeutics Center, Institute of Structural Biology, 85764 Neuherberg, Germany; Ludwig-Maximilians-University of Munich, Gene Center Munich and Department of Biochemistry, 81377 Munich, Germany; Ludwig-Maximilians-University of Munich, Gene Center Munich and Department of Biochemistry, 81377 Munich, Germany; Technical University of Munich, TUM School of Natural Sciences, Bavarian NMR Center and Department of Bioscience, 85748 Garching, Germany; Helmholtz Munich, Molecular Targets and Therapeutics Center, Institute of Structural Biology, 85764 Neuherberg, Germany; Technical University of Munich, TUM School of Natural Sciences, Bavarian NMR Center and Department of Bioscience, 85748 Garching, Germany; Helmholtz Munich, Molecular Targets and Therapeutics Center, Institute of Structural Biology, 85764 Neuherberg, Germany; Technical University of Munich, TUM School of Natural Sciences, Department of Bioscience, 85748 Garching, Germany; Technical University of Munich, TUM School of Natural Sciences, Department of Bioscience, 85748 Garching, Germany; Ludwig-Maximilians-University of Munich, Gene Center Munich and Department of Biochemistry, 81377 Munich, Germany; Technical University of Munich, TUM School of Natural Sciences, Bavarian NMR Center and Department of Bioscience, 85748 Garching, Germany; Helmholtz Munich, Molecular Targets and Therapeutics Center, Institute of Structural Biology, 85764 Neuherberg, Germany; Technical University of Munich, TUM School of Natural Sciences, Department of Bioscience, 85748 Garching, Germany; Ludwig-Maximilians-University of Munich, Gene Center Munich and Department of Biochemistry, 81377 Munich, Germany; Technical University of Munich, TUM School of Natural Sciences, Bavarian NMR Center and Department of Bioscience, 85748 Garching, Germany; Helmholtz Munich, Molecular Targets and Therapeutics Center, Institute of Structural Biology, 85764 Neuherberg, Germany

## Abstract

Biogenesis of small interfering RNAs (siRNA) in *Drosophila melanogaster* involves the processing of double-stranded RNA (dsRNA) by Dcr-2 with Loqs-PD/R2D2 and Ago2. Here, we show that Loqs-PD and Ago2 are found in biomolecular condensates *in vivo* and display liquid–liquid phase separation *in vitro*. The phase separation of Loqs-PD depends on the RNA-binding capability of its double-stranded RNA-binding domains and is further modulated by the preceding N-terminal region. An intrinsically disordered region in Ago2 (Ago2^IDR^) forms condensates in the presence of RNA *in vitro*. Combining NMR spectroscopy and mutational analysis, we show that Ago2^IDR^/RNA condensates are fluid, with significant polypeptide backbone flexibility, and are stabilized by a dense network of interactions involving arginine and aromatic side chains. Co-partitioning of Loqs-PD into Ago2^IDR^/dsRNA condensates depends on its ability to bind RNA. An RNase III enzyme can act on Ago2^IDR^/dsRNA condensates and reduce phase separation. Our results indicate that the unique features of the Ago2 IDR, which are broadly conserved in arthropods, drive biomolecular condensate formation, suggesting that phase separation plays a role in siRNA processing in *Drosophila*, potentially tuning the efficiency of dsRNA-mediated antiviral defense.

## Graphical Abstract

**Graphical Abstract:**
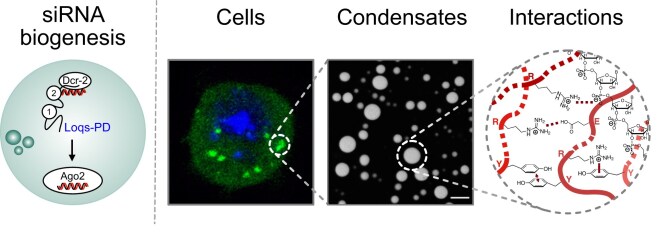

## Introduction

**Figure 1.**
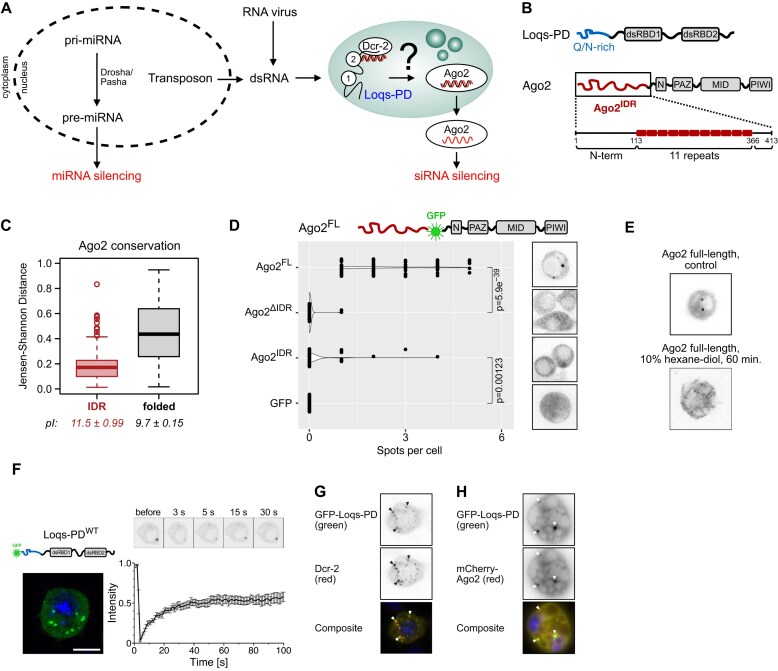
Loqs-PD forms MLOs in cells. (**A**) Schematic overview of *D. melanogaster* small RNA biogenesis, depicting how dsRNA is processed by Loqs-PD, Dcr-2, and Ago2 and additionally suggesting MLOs formation. (**B**) Domain structure of Loqs-PD and Ago2. Loqs-PD contains an N-terminal Q/N-rich region (blue), and Ago2 contains a repetitive and Q-rich IDR (red, Ago2^IDR^). (**C**) The extent of evolutionary conservation within a collection of arthropod Ago2 protein sequences, expressed as the Jensen-Shannon difference to a background distribution with absolute conservation represented as 1.0, differs between the disordered region and the folded part. Despite the much higher sequence variability, the very basic nature (high pI) within the IDR has been retained during arthropod evolution (see also Supplementary Fig. S4 for details). (**D**) GFP-Ago2 fusion proteins can form condensates in living cells. For this, the N-terminal IDR is necessary and partially sufficient (*P*-values: Wilcox rank-sum test). (**E**) The spots seen in cells expressing the full GFP-Ago2 fusion protein disappear upon treatment with 10% 1,6-hexanediol, indicating that condensation is reversible. (**F**) Loqs-PD forms cytoplasmic spots [scale bar 5 μm; DNA was stained with Hoechst 33 342 (shown in blue)]. Fluorescence recovery after photobleaching (FRAP) experiments demonstrate that the spots show liquid-like properties; top: sample images from a time series, bottom: quantitative analysis (±SE, *n* = 11) suggests that the mobile fraction of GFP-Loqs-PD within the spots is ∼50%. (**G**) Representative confocal microscopy images of immunostaining of inducible FLAG-Dcr-2 cells transfected with GFP-Loqs-PD wild-type. Cells were induced with 250 μM CuSO_4_ to express Dcr-2. Dcr-2 was stained by anti-FLAG coupled to Alexa Fluor 594 (red), and Loqs-PD was stained by anti-GFP coupled to Alexa Fluor 488 (green). Arrowheads highlight the colocalization of Loqs-PD and Dcr-2. DNA was stained with DAPI and is shown in blue in the composite. (**H**) Representative confocal microscopy images of cells transfected with GFP-Loqs-PD (green) and mCherry-Ago2 (red). Arrowheads highlight the colocalization of Loqs-PD and Ago2. DNA was stained with DAPI and is shown in blue in the composite.

Small RNA silencing post-transcriptionally regulates gene expression and mediates host defense against viral infections and transposable elements [1, 2]. In *Drosophila melanogaster*, RNA silencing is directed by microRNAs (miRNAs) (gene regulation) or small interfering RNAs (siRNAs) (defense against viruses and transposons) derived from longer, double-stranded (ds) precursors by nucleolytic processing through Dicer proteins in complex with smaller, double-stranded RNA-binding domain (dsRBD)-containing proteins, such as R2D2 and Loqs [3, 4]. The guide strand, which determines the messenger RNA (mRNA) targets for the RNA-induced silencing complex (RISC), is loaded into an effector protein of the Argonaute family [5–7]. miRNAs are processed by Dcr-1 with the help of the B-isoform of Loqs (Loqs-PB) and preferentially loaded into Ago1 [8]. Silencing then occurs in association with the protein GW182 and is localized in P-bodies, a cytoplasmic membrane-less organelle (MLO) that serves as a center for RNA turnover. While P-body formation is not essential for miRNA silencing, it could provide a kinetic advantage or sequester specific messenger ribonucleoproteins (mRNPs) [9]. In contrast, loading of Ago2 occurs via a specific RISC loading complex consisting of either Dcr-2/R2D2 [10] or Dcr-2/Loqs-PD (summarized in Fig. 1A) [11]. For the Dcr-2/R2D2 RLC, this is aided by the formation of cytoplasmic foci called D2-bodies [12], possibly enhanced by multimeric interactions between Dcr-2 and Taf11 [13].

MLOs have been shown to form through liquid–liquid phase separation (LLPS) and can allow the spatiotemporal organization of biochemical processes such as stress response and RNA splicing in cells [14, 15]. It has been proposed that intrinsically disordered regions (IDRs) in proteins, enriched in polar and aromatic residues (“stickers”), drive phase separation by forming a network of transient interactions [16]. Interspersed amino acids that do not participate in these interactions (“spacers”) weaken the network to prevent irreversible aggregation. A similar concept has been suggested for phase separation involving folded protein domains separated by disordered linkers [14, 17–20]. Phase separation can be driven by specific residue types such as tyrosines and arginines, which can mediate hydrophobic and electrostatic interactions, stabilizing the dense phase [21–24]. However, depending on the molecules and amino acids involved, mutational analysis, NMR spectroscopy, and molecular dynamics simulations have shown complex interaction networks involving various types of amino acids [23, 25–28]. Nucleic acids, particularly RNA, are well suited to participate in phase creation by serving as a polymeric, multivalent scaffold for interactions with proteins harboring RNA-binding domains, which often occur in multiple copies [29–31]. In addition, glutamine-rich IDRs can mediate LLPS by forming multivalent interactions involving the backbone and side chain functional groups [32–34]. In contrast to *Drosophila* Ago1, which mediates miRNA silencing and can partition into P-bodies by associating with GW182, the siRNA-effector *Drosophila* Ago2 has so far not been linked to biomolecular condensates. Interestingly, *Drosophila* Ago2 contains a highly repetitive, basic, and glutamine-rich N-terminal region (Ago2^IDR^), predicted to be intrinsically disordered (Fig. 1B). While the sequence of the Ago2^IDR^ is highly variable among related species, it nonetheless retains a strongly basic isoelectric point (pI = 11.5 ± 0.99) (Fig. 1C). Furthermore, the glutamine-richness and prion-like features are evolutionarily conserved [35, 36]. Finally, our previous work has demonstrated that the two dsRBDs of Loqs-PD bind RNA independently of each other, potentially leading to a dynamic meshwork of Loqs-PD/dsRNA interaction [11]. Given the occurrence of condensates in the siRNA and miRNA pathways and the unique presence of the Ago2^IDR^ in *Drosophila* (Fig. 1A), we wondered if the siRNA biogenesis factors Ago2 and Loqs-PD form condensates, which might contribute to efficient loading and siRNA processing in *Drosophila*.

Here, we show that Ago2, Loqs-PD, and RNA can form condensates with a liquid-like character *in vivo* that require dsRNA binding by Loqs-PD. *In vitro* experiments show that Ago2^IDR^ can phase-separate, governed by electrostatic interactions of arginines with nucleic acids and an extensive network of protein side-chain interactions within the dense phase. Strikingly, Loqs-PD can partition into the Ago2–dsRNA coacervates, but this activity requires the presence of dsRNA and dsRNA binding of Loqs-PD. Importantly, dsRNAs can be cleaved by an RNase III enzyme in the presence of Ago2–dsRNA condensates *in vitro*, reversing phase separation. Our data suggest that the availability of a dsRNA substrate may trigger the coacervation of Ago2, Loqs-PD, and dsRNA. Such condensates could provide a specific and efficient environment for siRNA biogenesis.

## Materials and methods

### Analysis of Ago2 protein sequences in extant species

We selected 45 species that together cover many of the insects but also include crustaceans. We included 12 *Drosophila* species to allow for resolution of recent divergence as well. Sequences were retrieved manually via BLAST search with the *D. melanogaster* protein sequences, each time limiting the search for the intended species. We note that the repetitive and potentially heterogeneous nature (= inter-individual variations within a species) of the Ago2 N-terminal region also poses a challenge to genome assemblies. We only considered full-length variants that represented the longest isoform reported for a given species and that harbor an initiating methionine. Subsequently, a multiple sequence alignment was created with T-coffee (tcoffee.crg.eu). For Ago2, the beginning of the conserved sequence could be derived from the multiple sequence alignment and was distinct from a more variable N-terminal part. The sequences were thus split into the N-terminal (non-conserved) and C-terminal (conserved) regions for separate analysis. We created a new multiple-sequence alignment for only the conserved region (again with T-coffee). Ad-hoc R! scripts were used to calculate and display pI-values and amino acid frequencies for the corresponding N-terminal and folded protein regions. The approach of Capra and Singh [37] was used to calculate the Jensen-Shannon distance to the background BLOSUM63 distribution with the windowing feature enabled at ±3 AA. Alignments were made for the full-length sequences and the N- and C-terminal sections separately, then submitted to https://compbio.cs.princeton.edu/conservation/score.html. The circular evolutionary tree diagram was created for the listed species with the help of phyloT v2 and is based on the NCBI Taxonomy and Genome Taxonomy databases.

### Cell culture

*Drosophila melanogaster* S2 cells were cultured in Schneider’s medium (Bio&Sell) with 10% fetal bovine serum (FBS, Sigma) and Penicillin/Streptomycin (Thermo Fisher Scientific) at 25°C. The cells were split weekly by diluting them 1:10 into fresh medium. The inducible Loqs in the *r2d2* ko cell line (Clone K54) and the inducible Dcr-2 cell line were generated by genome editing at the start codon of the protein of interest via a PCR-based CRISPR/Cas9 protocol developed in the Förstemann lab [38, 39]. For transient transfections, the specific plasmids were transfected with a concentration of 500 ng plasmid in 500 µl cell culture well (24-well plate) at a cell culture density of 1.5 × 10^6^ cells/ml, and the cells were examined at day 3 post-transfection. For the treatment with 1,6-hexanediol, the cells were transfected with GFP-Loqs-PD constructs 3 days before the treatment, while for GFP-Ago2, cell lines with stable expression were employed. On treatment day, the cells were split 1:5 into fresh medium containing 10% 1,6-hexanediol and subsequently examined via microscopy.

### Estimation of Loqs-PD concentration in one *Drosophila* cell

To estimate the concentration of Loqs-PD in one cell, the total protein of a counted number of S2 cells was isolated by direct boiling in SDS sample buffer. The volume corresponding to 2 × 10^4^ was loaded on an sodium dodecyl sulfate–polyacrylamide gel electrophoresis (SDS–PAGE) gel along with recombinant Loqs protein as calibration standards, transferred to a PVDF membrane and incubated with anti-Loqs antibody [11], and the chemiluminescence signal was recorded with a GE Amersham Imager 600 and quantified using the Fuji MultiGauge software. We estimated the size of our S2 cells and nucleus based on our microscopy images, assumed both to be spherical, and approximated the cytoplasmic volume as the difference of total cell volume and nuclear volume. Further details are given in the supplementary information and Supplementary Table S2.

### Immunostaining

For the immunostaining of the cells, cells were harvested at 5500 g for 10 min and washed once with 1× phosphate buffered saline (PBS). The cells were applied to a poly-l-lysine slide for 45 min to settle down and adhere to the surface, then fixed with 1× PBS + 3.6% formaldehyde for 3 min. Subsequently, the cells were washed for 10 min with 1× PBS + 1% Triton and afterward blocked 2 × 40 min in 1× PBS + 1% Triton + 1% BSA (bovine serum albumin). The cells were incubated with the primary antibody diluted in 1× PBS + 1% Triton + 1% BSA overnight in the cold room. The next day, cells were washed 3× for 1 h with 1× PBS + 1% Triton + 1% BSA and incubated with the secondary antibody (coupled to a fluorophore) overnight in the cold room. To avoid fading of the fluorophore, the incubations with the fluorophore were carried out in the dark (covered with aluminum foil). On day 3, the cells were washed 3× for 1 h with 1× PBS + 1% Triton + 1% BSA and then covered with mounting medium containing DABCO (1,4-diazabicyclo [2.2.2] octane) to prevent fading of the fluorescent signal and DAPI (4′,6-diamidino-2-phenylindole) to stain the DNA.

### Live cell imaging and FRAP assay

Imaging of GFP-Loqs-PD condensates in S2 cells and FRAP assays was performed using a Zeiss LSM710 confocal laser scanning microscope with a 63× oil immersion lens. Imaging of GFP-Ago2 condensates in S2 cells was performed using a Leica SP8 confocal laser scanning microscope with a 63× oil immersion lens. For live cell microscopy, the cells were placed in a chambered polymer coverslip at least 2 h before the microscopy session or the day before for FRAP assays so that the cells tightly adhere to the bottom of the slide. For bleaching the GFP-Loqs-PD condensates during the FRAP assay, a circular region of interest (ROI) was defined around the condensate and bleached at a wavelength of 488 and 561 nm with full laser power. After the recording of three scans, the ROI was bleached, and the fluorescence recovery was recorded. The images were processed with Fiji (ImageJ), and the FRAP assays were analyzed using the Stowers-ImageJ-Plugins.

### Protein expression and purification

The plasmids encoding Loqs-PD and Ago2^IDR^ sequences were cloned into a pETM11 or pET24 vector containing a His_6_-tag or His_6_-GB1 tag. Various Loqs-PD and Ago2 constructs were additionally cloned containing a fluorescent tag (monomeric GFP for Loqs-PD or mCherry for Ago2^IDR^) linked to the protein by a GS linker. For mutations of the repetitive sequence within Ago2 (Ago2^4-repeat^), gene fragments were ordered from Integrated DNA Technologies (IDT Europe GmbH) and cloned in a pET24 vector. All constructs are listed in Supplementary Table S3.

Recombinant proteins were expressed in *Escherichia coli* BL21 (DE3) cells. Proteins with fluorescent tags or those used for phase separation experiments were expressed in LB medium. For proteins used in NMR experiments (^15^N labeled or ^15^N,^13^C labeled), M9 minimal medium supplemented with 1 g/l ^15^NH_4_Cl and 2 g/l glucose or 1 g/l ^15^NH_4_Cl and 2 g/l [U-^13^C]-glucose was used. The bacterial cells were grown at 37°C to an OD_600_ of 0.8 and subsequently induced with 1.0 mM isopropyl-β-d-thiogalactopyranosid (IPTG). The proteins were expressed at 18°C overnight. The cells were harvested (7808 × *g*) and resuspended in 50 mM Tris, pH 8.0, 500 mM NaCl, and 10 mM imidazole (supplemented with lysozyme, 1 mg/ml DNase, 2 mM MgSO_4_, and protease inhibitor). After lysis of the cells using a French press and centrifugation (38 759 × *g*, 1 h), the cleared lysate was added to Ni–NTA resin, washed with 2 M NaCl, and eluted with 500 mM imidazole.

For all Ago2 constructs, the His_6_-tag was cleaved with His-tagged TEV protease at this stage of the purification (4°C, overnight). The protein was further purified by removing the cleaved His tag, uncleaved protein, and TEV protease from the desired protein on a second Ni–NTA column. The proteins were subsequently purified by ion-exchange chromatography on HiTrap SP or HiTrap Q columns (Cytiva) (20 mM Tris, pH 8.0, gradient from 0 to 1 M NaCl in 10 column volumes) followed by size-exclusion chromatography on a HiLoad 16/600 Superdex 75 column (GE Healthcare) (20 mM sodium phosphate, pH 6.5, 150 mM NaCl). Due to aggregation or phase separation of Loqs-PD proteins at low salt concentration, ion-exchange chromatography on HiTrap SP or HiTrap Q columns (Cytiva) (20 mM Tris, pH 8.0 or 20 mM sodium phosphate, pH 6.5, gradient from 0 to 1 M NaCl in 10 column volumes) was performed on proteins still containing the His_6_-GB1 tag. The His_6_-GB1 tag was then cleaved with His_6_-tagged TEV protease at 4°C overnight, and the protein was further purified by removing the cleaved His_6_-tag, uncleaved protein, and TEV protease from the desired protein on a second Ni–NTA column. In a last step, the proteins were purified by size-exclusion chromatography on a HiLoad 16/600 Superdex 75 or 200 column (GE Healthcare) (20 mM sodium phosphate, pH 6.5, 500 mM NaCl).

For preparation of the dense phase sample of Ago2^4-repeat^, the protein was mixed at high concentrations (3 mM) with heparin sodium salt (Sigma–Aldrich) as a nucleic acid mimetic (stock of 20 mg/ml) or 21 bp hairpin DNA (stock of 10 mM, IDT). The cloudy solution was centrifuged for 5 min to yield the dense phase at the bottom. More heparin or double-stranded DNA (dsDNA) was added to the supernatant until the solution did not turn cloudy anymore. This procedure was repeated to yield sufficient volume to transfer the solution to a 3 mm NMR tube or 3 mm NMR Shigemi tube. The sample was either prepared with only ^13^C^15^N-labeled protein or as a mixture of ^15^N,^13^C-, and unlabeled protein (1:5). Procedures to estimate the concentration of protein and DNA in the dense phase and to prove the presence of heparin in the dense phase are described in the Supplementary methods.

### *In vitro* transcription

Twenty-one base pair hairpin dsRNA was *in vitro* transcribed from a single-stranded DNA (ssDNA) template containing the T7 promoter sequence (Supplementary Table S1, IDT Europe GmbH) using in-house produced 3 μl of T7 RNA polymerase (production protocol in Supplementary methods) at 37°C for 3 h (0.64 μM DNA template, 0.64 μM T7 primer, 50 mM MgCl_2_, 8 mM of each rNTP, 5% PEG8000, 10% dimethyl sulfoxide (DMSO), and 1× transcription buffer). The reaction mix was purified on a DNAPac PA200 anion exchange column (22 × 250 mm, Thermo Fisher Scientific) by high-performance liquid chromatography (12.5 mM Tris, pH 8.0, 6 M urea, gradient from 0 to 0.5 M sodium perchlorate) at 85°C. Before use, the RNA was heated to 95°C for 2 min and snap-cooled on ice.

### Fluorescent electrophoretic mobility shift assay (EMSA)

For qualitative analysis of differences in RNA binding of Loqs-PD^WT^ and Loqs-PD^1,2-mut^, the 21 bp hairpin dsRNA was fluorescently labeled by ligation with pCp-Cy5 to the 3′ end of the dsRNA using T4 RNA ligase, and the reaction mix was purified using a spin column kit from Norgen Biotek Corp. Four hundred nanomolar 21 bp hairpin dsRNA in 20 mM sodium phosphate, pH 6.5, 150 mM NaCl, and 15% glycerol was incubated with increasing concentrations of Loqs-PD^WT^ (100, 200, 400, 800, 1200, 1600, and 3200 nM) or Loqs-PD^1,2-mut^ (400, 800, and 3200 nM) for 15 min. Gel electrophoresis was performed on an agarose gel [1% (w/v) agarose] in 1× TBE buffer at 40 V for 3 h. For detection, an Azure Sapphire biomolecular imager at 649 nm was used.

### *In vitro* phase separation assays

For Ago2, phase separation was initiated by the addition of nucleic acids (dsRNA, dsDNA, single-stranded RNA (ssRNA), and ssDNA) or nucleic acid mimetics (heparin). Used oligonucleotides are listed in Supplementary Table S1. The DNA was ordered from Eurofins Genomics or IDT. Before use, the dsDNA and dsRNA were heated to 95°C for 2 min and snap-cooled on ice. Salt concentrations of the buffer (20 mM sodium phosphate, pH 6.5, X mM NaCl) and temperature (25°C or 5°C) were varied to analyze the effect on the phase separation behavior. Frequently used buffers for phase separation assays are listed in Supplementary Table S4. For Loqs-PD, the stock solution (200 μM) was stored at 20 mM sodium phosphate, pH 6.5, and 500 mM NaCl to avoid phase separation and was further concentrated directly before usage if necessary. Phase separation was induced by reducing the salt concentration or adding 21 bp hairpin dsRNA. Phase separation of different Ago2 and Loqs constructs at varying conditions (protein concentration, salt concentration, RNA concentration, and PEG8000 concentration) was assessed by microscopy and turbidity measurements. For turbidity measurements, 10 μl sample was mixed at the respective salt, protein, and RNA concentrations, and the OD_600_ was measured on a NanoDrop 2000 (Thermo Fisher Scientific). The effect of RNase A (100 μg/ml) on phase separation of Ago2^IDR^ (50 μM) and 21 bp hairpin dsRNA (50 μM) was assessed by microscopy, and the cleavage was monitored by gel electrophoresis on a polyacrylamide gel (12%, containing 8 M urea) in 1× TBE buffer. Sedimentation assays were performed to quantify phase separation propensities and the effect of temperature on this propensity. Phase separation of the constructs (Ago2^4-repeat^ WT and mutants, Ago2^N-term^ WT and mutants) (800 μM) was induced by incubating with 2 mg/ml heparin at 25°C or 5°C for 5 min. Subsequently, the samples were centrifuged at the respective temperatures for 5 min (21 130 × *g*), and the absorption of the supernatant at 280 nm was measured on a NanoDrop 2000 to estimate the protein concentration in the dense and dilute phases. All measurements were performed in triplicates. For Ago2^4-repeat^ (800 μM), 1 μl sample before the addition of 2 mg/ml heparin and 1 μl of the described supernatant at 25°C or 5°C were diluted in 20 μl buffer (20 mM sodium phosphate, pH 6.5, 50 mM NaCl) and analyzed via SDS–PAGE.

### Microscopy of *in vitro* condensates

The effect of protein concentration, NaCl concentration (50, 100, and 150 mM), pH (5.5, 6.5, 7.5), ammonium acetate concentration (500 mM), 1,6-hexanediol (5%), and PEG8000 (1%–5% PEG) on phase separation propensity was tested by phase contrast or confocal microscopy. To minimize the effect of the fluorescent tag, nonlabeled proteins are mixed with fluorescently tagged proteins in fluorescent experiments (0.5 μM of mCherry- or GFP-tagged proteins, captions state the total protein concentration). Additionally, condensates of untagged proteins were stained with the fluorophore sulforhodamine B (0.2 μM). For co-condensation assays of Ago2^IDR^, 21 bp hairpin dsRNA, and various Loqs-PD constructs, Ago2^IDR^ (45 μM Ago2^IDR^, 5 μM Ago2^IDR^-mCherry, 20 mM phosphate, pH 6.5, 50 mM NaCl) was mixed with 25 μM 21 bp hairpin dsRNA to induce phase separation. Then, the Loqs-PD construct (45 μM Loqs-PD construct, 5 μM GFP-Loqs-PD construct) was added and examined by confocal microscopy. Bright-field microscopy was performed on an IM-3LD microscope from OPTIKA (60×). A circular chamber was built with medium-viscosity silicone grease on the glass slide.

Confocal fluorescence microscopy was performed on a Leica TCS SP8 confocal microscope using a 63× water immersion objective (1.2 NA) and a PMT detector. Samples were prepared by mixing the proteins at the desired protein and salt concentration. If required, 21 bp hairpin dsRNA was added. The samples were transferred to micro-well plates (ibidi, μ-Slide Angiogenesis Glass Bottom). Droplets formed by unlabeled proteins were stained with the fluorophore sulforhodamine B (0.2 μM), which was excited at 552 nm and detected from 565–700 nm. When labeled proteins were used, samples were excited with the 488 nm laser line (GFP) and 552 nm (mCherry), and imaged at 495–550 nm and 580 and 650 nm, respectively. If more than one fluorophore was present in the sample, both channels were recorded simultaneously. To rule out bleed-through, single-labeled controls were carried out, maintaining acquisition parameters constant. In all experiments, the pinhole was set to 1 Airy unit. Measurements were done at 24°C.

FRAP experiments were performed for GFP-Loqs-PD^FL^, GFP-Loqs-PD^ΔC^, and mCherry-Ago2^IDR^. Samples were prepared as previously described on PVA-passivated well plate chambers (ibidi, μ-slide Angiogenesis Glass Bottom). Each FRAP experiment consisted of 10 pre-bleaching frames, 10 bleaching frames followed by the recovering frames. Part of the droplets were bleached, and imaging time varied depending on the ROI and sample but was typically between 200 and 400 ms/frame. All data analysis was performed in ImageJ 2.1.0. [40]. For FRAP, the fluorescence intensity as a function of time for the bleached area, reference, and background was obtained using ImageJ 2.1.0, and the recovery of the bleached region was normalized against the background and the reference region. Data were fitted to a single exponential to obtain the half-time of recovery as shown before [41].

### Dynamic light scattering

For dynamic light scattering (DLS), 100 μl of sample (50 μM protein concentration for Ago2^IDR^, 100 and 1000 μM protein concentration for Ago2^4-repeat^, 20 mM sodium phosphate, pH 6.5, 50 mM NaCl, 2 mM DTT) were measured at 25°C on a DynaPro NanoStar (Wyatt). Before the experiment, the protein samples were centrifuged for 10 min at 21 130 × *g*. To measure phase-separated samples, 25 μM of 21 bp hairpin dsRNA or 0.2 mg/ml of heparin were added to induce phase separation. Ammonium acetate (200 mM) and higher heparin concentration (2 mg/ml) were added to analyze the effect on the hydrodynamic radius. To assess temperature effects, Ago2^4-repeat^ WT and mutants (100 μM) with 0.2 mg/ml heparin were measured at 25°C and after cooling on ice for 5 min. The data were analyzed using the DYNAMICS software package.

### NMR spectroscopy

All NMR samples (^15^N,^13^C, or ^15^N-labeled, as appropriate) were measured in NMR buffer (20 mM sodium phosphate, pH 6.5, 50 or 150 mM NaCl, 2 mM DTT) containing 10% (v/v) D_2_O at 25°C on 1200-, 950-, 900-, 800-, 600-, or 500-MHz Bruker Avance NMR spectrometers equipped with cryogenic triple-resonance gradient probes. All spectra were measured in 3 mm NMR tube. For nuclear Overhauser enhancement spectroscopy (NOESY) experiments of the dense phase (Ago2^4-repeat^ with heparin), a 3 mm Shigemi tube was used. Sample concentrations were chosen according to protein stability, expression yield, or phase separation behavior of the specific protein, as described in the figure legends. NMR spectra were processed with TOPSPIN3.5 (Bruker) or NMRPipe [42] and analyzed using NMRFAM-Sparky [43].

#### Chemical shift assignment

Protein backbone assignments for Ago2^N-term^ and Ago2^4-repeat^ were obtained from standard 3D heteronuclear HN(CA)CO, HNCO, HNCACB, and CBCA(CO)NH backbone experiments. Further side-chain resonances were assigned using CC(CO)NH, HCC(CO)NH, hCCH-TOCSY, and HcCH-TOCSY experiments [44, 45]. Secondary structure propensities were derived from the difference of ^13^C_α_ and ^13^C_β_ secondary chemical shifts Δδ^13^C_α_–Δδ^13^C_β_ [46–48]. For dense phase samples (prepared with heparin or 21 bp hairpin DNA), most assignments of the dilute phase could be transferred. To confirm and further improve assignments HNCACB and CBCA(CO)NH backbone experiments and hCCH-TOCSY and HcCH-TOCSY side chain experiments were recorded. For Loqs-PD, previous chemical shift assignments of Loqs^ΔNC^ were transferred [11]. Additionally, protein backbone assignments of the unstructured N-terminus were obtained, as described above.

#### Titrations

^1^H,^15^N HSQC spectra were measured after each addition of titrant, and the changes were visualized by calculating the chemical shift perturbation (CSP) based on the following equation: ${\mathrm{\Delta }}\delta = \sqrt {{\mathrm{\Delta }}\delta _{HN}^2 + {{( {\frac{{{\mathrm{\Delta }}{\delta }_N}}{{6.5}}} )}}^2}$ [49]. In the case of line-broadening of NMR signals the intensity of the peaks was used to assess the effect of binding.

#### Diffusion ordered spectroscopy

For 1D diffusion experiments [50] (stimulated echo) of Ago2^IDR^ (100 μM) before (non-phase separated) and after addition of 100 μM 21 bp hairpin dsRNA (biphasic) were measured (800 MHz ^1^H Larmor frequency). For 2D ^1^H,^13^C diffusion experiments (based on a 2D ^1^H,^15^N diffusion experiment [51]), Ago2^4-repeat^ (420 μM, non-phase separated), Ago2^4-repeat^ after addition of heparin (420 μM, 1 mg/ml heparin, biphasic), and Ago2^4-repeat^ with heparin (∼30 mM, ∼30 mg/ml heparin, dense phase) were measured (900 MHz ^1^H Larmor frequency). A linear diffusion gradient from 2%–98% over 8 (for 2D ^1^H,^13^C diffusion experiments) or 10 measurement points (for 1D diffusion experiments) was used. For the non-phase separated and the biphasic sample of Ago2^4-repeat^ a diffusion delay of 0.3 s and a gradient pulse of 0.003 s, for the non-phase separated sample and biphasic sample of Ago2^IDR^ a diffusion delay of 0.4 s and a gradient pulse of 0.004 s and for dense-phase sample of Ago2^4-repeat^ a diffusion delay of 0.5 s and a gradient pulse of 0.005 s was used. Data were analyzed using the Dynamics Center 2.4.8 software (Bruker).

#### Relaxation experiments

^15^N relaxation experiments [52] were recorded on a 500-MHz Bruker Avance NMR spectrometer (^15^N *T*_2_ for Ago2^N-term^), a 900-MHz Bruker Avance NMR spectrometer ({^1^H}-^15^N heteronuclear NOE, ^15^N *T*_1_ and *T*_2_ for Ago2^4rep^) and a 950-MHz Bruker Avance NMR spectrometer (^15^N *T*_1ρ_ for Ago2^4rep^ and heteronuclear NOE for Ago2^N-term^) at 25°C. For {^1^H}-^15^N heteronuclear NOE experiments, NOE values were determined from the peak height ratio of the experiment with and without proton saturation [53]. ^15^N *T*_1_, *T*_2_, and *T*_1ρ_ relaxation times were acquired from pseudo-3D HSQC experiments in an interleaved manner with nine relaxation delays for *T*_1_ (dilute phase: 50, 100, 200, 350, 600, 900, 1300, 1800, and 2600 ms, dense phase: 50, 100, 200, 350, 500, 800, 1100, 1500, and 2000 ms), *T*_2_ (dilute phase and dense phase: 16.96, 33.92, 101.76, 135.68, 169.6, 254.4, 305.28, 407.04, and 644.48 ms), and *T*_1ρ_ (dilute phase: 6, 24, 48, 72, 96, 160, 200, 300, and 400 ms, dense phase: 6, 24, 36, 48, 72, 96, 120, 160, and 180 ms). Residual relaxation rates were obtained by fitting the data to an exponential function using NMRFAM-Sparky [43] and fitting errors were calculated.

#### Nuclear Overhauser enhancement spectroscopy

2D ^1^H,^1^H NOESY experiments (Bruker library: noesygpph19, 900 MHz ^1^H Larmor frequency) for the non-labeled dilute phase (Ago2^4-repeat^ and heparin, respectively) and dense phase (Ago2^4-repeat^ with heparin) were recorded at NOE mixing times of 150 ms. 3D ^15^N-edited NOESY and ^13^C-edited NOESY-HMQC experiments of the dilute phase of Ago2^4-repeat^ (1 mM, PS-I buffer) were recorded at 950 MHz at NOE mixing times of 150 ms. Intermolecular contacts in the dense phase (Ago2^4-repeat^ with heparin) were identified via 3D *ω*_1_–^13^C, ^15^N-filtered *ω*_3_–^15^N-edited and *ω*_1_–^13^C, ^15^N-filtered, *ω*_3_–^13^C-edited NOESY-HSQC experiments, respectively, performed at 28.2 T (1.2 GHz ^1^H Larmor frequency) NOE mixing times of 150 ms. To assess the effect of spin diffusion *ω*_1_–^13^C, ^15^N-filtered *ω*_3_–^13^C-edited NOESY-HSQC were additionally measured at NOE mixing times of 80 and 30 ms, and 2D ^1^H,^1^H NOESY experiments were measured at mixing times of 10, 30, 50, 80, 120, 150, 200, 300, 400, and 500 ms (1.2 GHz ^1^H Larmor frequency). To distinguish between intra- and intermolecular NOEs, we employed an adiabatic 13C,15N double filter element as described previously [54, 55]. This filter suppresses signals from protons directly bonded to ^13^C or ^15^N nuclei, thereby allowing selective observation of NOEs involving protons from unlabeled interaction partners. In the filtered experiment, heteronuclear scalar couplings, ^1^J(^1^H,^13^C) and ^1^J(^1^H,^15^N), are not refocused during chemical shift evolution, as only signals from unlabeled molecules where such couplings are absent are observed. However, in practice, the filter may not operate with 100% efficiency, and signals from isotope-labeled protein may leak through. These unwanted cross peaks can lead to misinterpretation, as they may be incorrectly assigned as intermolecular NOEs. To identify such artifacts, we recorded a second version of the experiment with heteronuclear decoupling applied during the chemical shift evolution period. In this decoupled experiment, scalar couplings are refocused, and artifact signals from labeled protons appear as singlets, whereas in the non-decoupled (filtered) version, they typically show splitting due to scalar coupling. By comparing the two spectra, we identified intermolecular NOE cross peaks as singlets in both experiments, indicating that they arise from protons not coupled to ^13^C or ^15^N, and thus belong to the unlabeled interaction partner. Conversely, any cross peaks that showed splitting in the filtered spectrum but collapsed into singlets in the decoupled version were identified as originating from the labeled protein and excluded from further analysis.

NOE intensities from the *ω*_1_–^13^C, ^15^N-filtered, *ω*_3_–^13^C-edited NOESY-HSQC experiment (mixing time of 80 ms) of resolved cross peaks were evaluated to assess intermolecular contacts in the dense phase (Ago2^4-repeat^ with heparin). The intensities are normalized by correcting for the number of a given amino acid in the sequence. Note that the representation of intermolecular contacts is not complete since only well-resolved and unambiguously assigned NOE cross peaks have been analyzed.

## Results

### Ago2 and Loqs-PD form MLOs in cells

The presence of a disordered domain in the N-terminal region of Ago2 is deeply conserved (Fig. 1C and Supplementary Fig. S4C). We inserted fluorescent proteins (muGFP or mCherry) between the disordered domain and the folded part of the Ago2 protein to observe Ago2 in living cells. As controls, we also created a fusion protein that lacks the N-terminal IDR, one that lacks the folded part of Ago2, and finally, muGFP fused only to the linker necessary for cloning. We then established stable cell lines with expression levels roughly comparable with the endogenous Ago2 protein in Schneider-2 cells (Supplementary Fig. S1A). Full-length IDR-GFP-Ago2 protein readily formed spots of high local concentration in living cells. These spots were far less frequently observed in cells expressing the GFP-fusion protein lacking the IDR, and fusing only the IDR to GFP can be sufficient to induce an intermediate level of spots in a subset of cells (Fig. 1D). These spots were not well-rounded, indicative of some degree of maturation/hardening. However, treatment with hexanediol could dissolve the spots, indicating that the condensation was still reversible (Fig. 1E).

We previously demonstrated that Loqs-PD and Dcr-2 form an alternative RISC loading complex (aRLC) in our isolate of the *Drosophila* Schneider-2 (S2) cell line [11]. For the related Dcr-2/R2D2 RLC, the cytoplasmic D2-body is required for Ago2 loading, yet certain siRNA species remain Ago2-loaded in the absence of R2D2 [12]. This raises the question if there is a corresponding cellular context of alternative RISC loading. We transfected plasmids encoding monomeric muGFP-fusion proteins with Loqs-PD into cultured *Drosophila* S2 cells and analyzed the sub-cellular localization by confocal fluorescence microscopy. This revealed that GFP-Loqs-PD forms cytoplasmic “spots” of high local concentration (with an apparent size ∼0.2–0.4 μm in diameter, i.e. diffraction-limited) in addition to a lower level of uniformly distributed Loqs-PD (Fig. 1F and Supplementary Fig. S1B and C). Co-transfection of orthogonally tagged myc-Loqs-PB revealed a partial overlap, while myc-R2D2 appeared to form distinct but in certain cases, adjacent foci (Supplementary Fig. S1D). To assess the dynamic properties of these condensates, we conducted FRAP experiments with transfected cells. These experiments demonstrated that the condensates exhibit a partially liquid-like character, with about half of the GFP-Loqs-PD exchanging with the surrounding solution after ∼40 s (Fig. 1F), while the other half appeared immobile during our observation time of 100 s. Treatment of cells with 1,6-hexanediol, known to disrupt molecular interactions that stabilize the phase-separated condensates, nonetheless resulted in the complete disappearance of the spots (Supplementary Fig. S1E). We conclude that the spots form via a reversible condensation phenomenon rather than irreversible aggregation.

For the emergence of D2 bodies, the presence of R2D2 was reported to be essential [12]. In genome-edited S2 cells that lack R2D2 [11] (Supplementary Fig. S1F–H), the spot formation by transfected GFP-Loqs-PD was unchanged relative to wild-type cells; hence, R2D2 is not required for the formation of the GFP-Loqs-PD containing droplets (Supplementary Fig. S1H).

Furthermore, we transfected GFP-tagged Loqs-PD into a genome-edited S2 cell line that has inducible expression of Flag-tagged Dcr-2 (Supplementary Fig. S1I and J) and either left Dcr-2 uninduced or induced Flag-Dcr-2 expression. In both cases, GFP-Loqs-PD formed cytoplasmic condensates, hence Dcr-2 does not appear to be essential for the formation of this condensate. Nonetheless, co-immunostaining of induced Flag-Dcr-2 and GFP-Loqs PD revealed the extensive colocalization of Dcr-2 and Loqs-PD (Fig. 1G). Additionally, co-expression of Ago2 and Loqs-PD showed that both proteins can colocalize in cells (Fig. 1H).

### Loqs-PD can undergo phase separation *in vitro*

**Figure 2.**
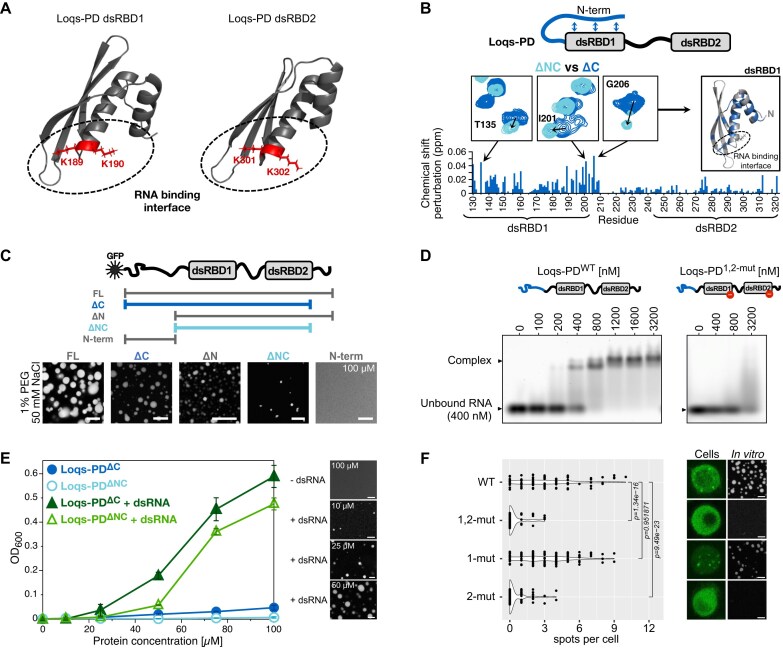
RBDs of Loqs-PD are essential for phase separation. (**A**) The RNA-binding interface of Loqs-PD is shown on the dsRBD1 and dsRBD2 structure (pdb: 5NPG, 5NPA). The RNA-binding site is highlighted, and key lysine residues are shown as sticks in red. (**B**) Intramolecular interactions of the N-terminal region and dsRBD1 in Loqs-PD. ^1^H–^15^N HSQC spectral comparison of Loqs-PD^ΔNC^ (cyan) and Loqs-PD^ΔC^ (blue) is shown for the key regions, while the overall CSP is plotted against all the residues in blue. CSP > 0.015 are marked on the dsRBD1 structure (pdb: 5NPG) in blue, the RNA-binding site is highlighted, and key lysine residues are shown as sticks. (**C**) Loqs-PD dsRBDs are essential for phase separation. Top: Schematic representation of the domain structure of Loqs-PD (N-terminal region, dsRBD1 and 2) with an N-terminal GFP tag. Bottom: Fluorescent microscopy images of those constructs (100 μM protein concentration, PS-I buffer, 1% PEG8000, scale bar 10 μm). (**D**) Qualitative comparison of binding of Loqs-PD^WT^ (left) and Loqs-PD^1,2-mut^ (right) to 21 bp hairpin dsRNA by fluorescent EMSA. Loqs-PD^1,2-mut^ contains KK to AA mutations in both dsRBDs that hinder the RNA binding. (**E**) RNA-dependent droplet formation of Loqs-PD. Turbidity assay (OD_600_, left) is performed with increasing concentrations of Loqs-PD^ΔC^ and Loqs-PD^ΔNC^ in the absence (circles, Loqs-PD^ΔC^: blue, Loqs-PD^ΔNC^: cyan) and presence (triangles, Loqs-PD^ΔC^: green, Loqs-PD^ΔNC^: grass green) of 21 bp hairpin dsRNA (25 μM RNA, PS-I buffer). Error bars represent the standard deviation of three replicates from distinct samples. Fluorescent microscopy is performed for Loqs-PD^ΔC^ (right) at various concentrations in the presence and absence of 21 bp hairpin dsRNA (25 μM RNA, PS-II buffer, scale bar 10 μm, 0.2 μM sulforhodamine dye). (**F**) Mutations of residues critical for RNA-binding abolish Loqs-PD spot formation. Left: Quantification of the number of spots observed per cell for various Loqs-PD constructs (*P*-values: Wilcox rank-sum test). Right: Fluorescent microscopy images in cells and *in vitro* (50 μM protein concentration, 25 μM RNA, PS-II buffer, 0.2 μM sulforhodamine dye). Loqs-PD^1,2-mut^ has a KK to AA mutation that hinders RNA binding in both dsRBDs, Loqs-PD^1-mut^ in dsRBD1 and Loqs-PD^2-mut^ in dsRBD2, respectively. Additional fluorescent microscopy images in cells are exemplarily shown in Supplementary Fig. S3B.

To assess the structural features of different regions in Loqs-PD, we generated several deletion mutants of Loqs-PD, namely Loqs-PD^ΔC^ (C-terminal region deleted), Loqs-PD^ΔN^ (N-terminal region deleted), Loqs-PD^ΔNC^ (N- and C-terminal regions deleted) and Loqs-PD^N-term^ (only N-terminal region) (Fig. 2A and Supplementary Fig. S2A) and performed NMR spectroscopy. Comparison of NMR fingerprint spectra of deletion mutants and full-length Loqs-PD^FL^ (Fig. 2B and Supplementary Fig. S2A) showed small but significant chemical shift differences for amides in dsRBD1 in the presence of the N-terminal region (Loqs-PD^ΔNC^ versus Loqs-PD^ΔC^), involving residues in the dsRNA-binding interface of dsRBD1 (Fig. 2B and Supplementary Fig. S2B top). This suggests that the N-terminal region may weakly interact with the dsRNA-binding surface of dsRBD1 and partially compete with RNA binding. This is consistent with the observation that the presence of the N-terminal region weakens the binding of Loqs-PD to long, blunt-ended dsRNA [56].

To reconstitute full-length Loqs-PD (Loqs-PD^FL^) condensates *in vitro*, we tested various conditions on untagged and GFP-tagged Loqs-PD proteins using phase contrast and fluorescence microscopy. *In vitro* phase separation was favored at high protein concentration (100 µM) and low salt concentration (50 mM). Dissolution of the droplets at higher salt concentrations indicates the phase separation is driven mainly by electrostatic interactions (Fig. 2C and Supplementary Fig. S2C). The addition of 5% PEG to mimic the crowded environment in the cell induces phase separation at low protein concentrations (1 and 10 μM) and ionic strength at physiological-like levels (Supplementary Fig. S2D). This corresponds well to the low micromolar concentration of Loqs-PD in S2 cells (Supplementary Fig. S1J). FRAP experiments (fluorescence recovery, *T*_1/2_ ∼ 46 s) and the observation of droplet fusion demonstrate the liquid-like character of the *in vitro* condensates (Supplementary Fig. S2E and F).

To delineate the regions involved in droplet formation, we examined deletion constructs of Loqs-PD (± GFP-tag) (Fig. 2C and Supplementary Fig. S2G). *In vitro* condensate formation was observed—though to a variable extent—for all deletion constructs retaining the dsRBDs, suggesting the dsRBDs are indispensable for the phase separation even in the absence of dsRNA (Fig. 2C and Supplementary Fig. S2G). The phase separation was further enhanced by the presence of the N-terminal region.

To evaluate the importance of the two dsRBDs and the potential role of the N-terminal intramolecular interaction for phase separation, we examined the effect of RNA binding (Fig. 2D). The addition of a 21 bp hairpin dsRNA (Supplementary Table S1) to Loqs-PD^ΔC^ significantly enhanced phase separation (Fig. 2E), as seen by droplet formation and increased turbidity at 10 µM Loqs-PD. NMR titrations and microscopy at different salt concentrations confirmed the salt sensitivity of RNA-induced phase separation (Supplementary Fig. S2H), indicating that electrostatic interactions are important for condensate formation. This is reflected by line-broadening for amide NMR signals in the N-terminal region and the linker connecting the two dsRBDs upon adding dsRNA for the conditions where phase separation occurs (lowest and physiological-like salt concentration, 50 and 150 mM, respectively, Supplementary Fig. S2H). Our previous study showed that the linker does not contribute to RNA binding [11]. However, we observe weak RNA interactions by the unstructured N-terminal region of Loqs-PD at lower salt concentrations that are presumably electrostatically driven (Supplementary Fig. S2B bottom). Taken together, we identified NMR signal changes in the N-terminal and the dsRBD linker regions upon addition of RNA under phase separation conditions. Consistently, the Loqs-PD constructs lacking the N-terminal region show decreased phase separation in the presence of dsRNA (Fig. 2E and Supplementary Fig. S2G). This could be rationalized by increased intermolecular protein–protein interactions of the N-terminal region in the dense phase, which are further enhanced in the presence of RNA by the release of the N-terminal region from the transient intramolecular interaction with dsRBD1 (Fig. 2B, C, and E, and Supplementary Fig. S2B).

To explore the role of RNA binding by Loqs-PD for spot formation, we designed structure-based point mutations [11] that abolish the binding of dsRNA to dsRBD1, dsRBD2, or both dsRBDs of Loqs-PD (Fig. 2A and D and Supplementary Fig. S3A). We found that RNA binding to dsRBD2 was particularly important for the formation of the condensate in cells and *in vitro* (Fig. 2F and Supplementary Fig. S3B–F), while RNA interactions with dsRBD1 seem dispensable for the formation of cytoplasmic spots. Rather, they endow the condensate with a liquid-like state (Supplementary Fig. S3D). Taken together, we conclude that RNA binding involving the Loqs dsRBDs is essential and that the N-terminal region enhances phase separation.

### Ago2^IDR^ undergoes RNA-mediated phase separation involving electrostatic interactions

The N-terminal region of Ago2 is predicted to be intrinsically disordered and has a prion-like sequence composition (Supplementary Fig. S4A and B). It has previously been recognized that the sequence of the Ago2^IDR^ is highly variable among related species and even among different *D. melanogaster* isolates, while the catalytic and folded part of Ago2 is well conserved [35, 36]. We refined this analysis with a selection of the arthropod Ago2 protein sequences for 45 species (Fig. 1C and Supplementary Fig. S4C and D) and concluded that the basic and disordered N-terminal region has been maintained from crustaceans to flies. A compositional bias toward a high glutamine and/or arginine content can be clearly discerned (Supplementary Fig. S4E top), and the N-terminal disordered segment is consistently more basic than the catalytic part of Ago2 (Supplementary Fig. S4E middle, bottom). We conclude that the presence of a basic, glutamine-rich and disordered N-terminal domain in Ago2 is deeply conserved, despite the fact that RNAi-related genes show a higher rate of adaptive protein evolution [57].

**Figure 3.**
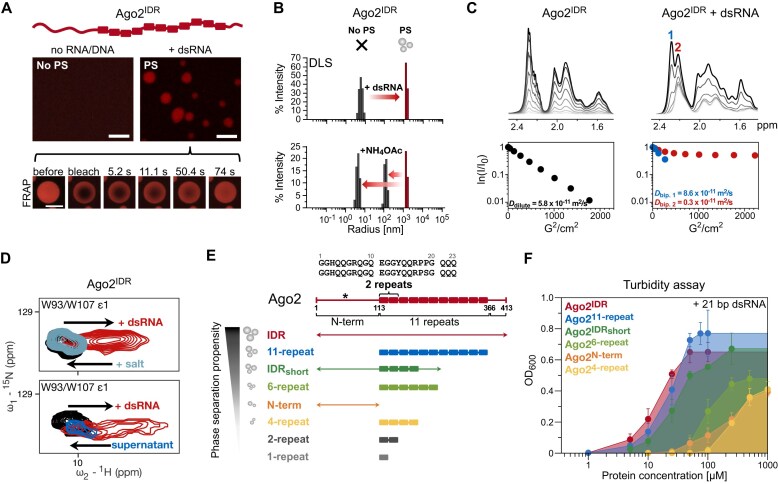
Ago2^IDR^ forms droplets upon the addition of nucleic acids. (**A**) RNA-induced droplets of Ago2^IDR^ (50 μM, PS-I buffer) in the presence of fluorescein-labeled bantam dsRNA (scale bar 10 μm). Liquid-like properties of Ago2^IDR^/21 bp hairpin dsRNA condensates are confirmed by time series of FRAP experiments (bottom, 50 μM protein, 25 μM RNA, PS-I buffer, bleach spot radius: 3.2 μm, droplet radius: 6.1 μm, scale bar 10 μm). The sequences of the bantam dsRNA and 21 bp hairpin dsRNA are shown in Supplementary Table S1. (**B**) Particle size changes upon the addition of 21 bp hairpin dsRNA to Ago2^IDR^ observed by DLS. Top: Particle sizes of Ago2^IDR^ (50 μM, PS-I buffer) before (black) and after adding 21 bp hairpin dsRNA (25 μM, red). Bottom: Particle sizes after addition of 200 mM ammonium acetate to the Ago2^IDR^/dsRNA sample. (**C**) Distinct diffusion rates of Ago2^IDR^ (100 μM, PS-I buffer) without (dilute phase, left) and with 21 bp hairpin dsRNA (100 μM, biphasic, right) by 1D NMR diffusion experiments (*D* = 0.4 s, *d* = 0.004 s, 800 MHz ^1^H Larmor frequency), corresponding to the size changes. (**D**) NMR spectral changes of Ago2^IDR^ upon phase separation. Superimposed tryptophan indole e1 peak in the ^1^H–^15^N HSQCs of Ago2^IDR^ (100 μM, PS-I buffer) without (black, dilute phase), with 21 bp hairpin dsRNA (100 μM, biphasic, red) and after addition of 100 mM NaCl (light blue, top) or the supernatant after spinning out the droplets by centrifugation (blue, bottom). Full spectra are shown in Supplementary Fig. S5I and J. (**E**) Schematic representation of different sub-constructs of Ago2^IDR^ (asterisk denotes the region with sequence polymorphism, details in Supplementary Fig. S4A) and their phase separation propensity based on turbidity assays (Fig. 3F). (**F**) Phase separation propensity of different sub-constructs of Ago2^IDR^ in the presence of 21 bp hairpin dsRNA (25 μM) by a turbidity assay (OD_600_) with increasing protein concentrations in PS-I buffer. Error bars represent the standard deviation.

We investigated the properties of the Ago2^IDR^*in vitro* (Supplementary Fig. S4A, for amino acid sequence details) and observed no phase separation of Ago2^IDR^ on its own. However, Ago2^IDR^ readily formed droplets in the presence of nucleic acids or nucleic acid mimetics such as heparin, suggesting that coacervation involves the positively charged Ago2^IDR^ and a negatively charged polyanion such as nucleic acids (Fig. 3A, Supplementary Figs S4F and S5A–E, and Supplementary Table S1). Droplet formation was observed at protein concentrations as low as 1 μM (Supplementary Fig. S5A). FRAP experiments, which showed fast fluorescence recovery within the condensed phase (*T*_1/2_ ∼ 2.7 s), and the observation of dynamic droplets that could fuse together confirmed the liquid-like character of the condensates (Fig. 3A and Supplementary Fig. S5F and G). Compared to Loqs-PD, the fluorescence recovery and fusion of *in vitro* generated droplets were significantly faster (Supplementary Figs S2E and F and S5F and G). While the specific type of nucleic acid seems less important (Supplementary Fig. S5B), a certain nucleotide length was required to induce phase separation (Supplementary Fig. S5D). As expected for electrostatic-driven interaction, droplet formation was favored at lower salt concentrations and was sensitive to ammonium acetate (Fig. 3B and Supplementary Fig. S5C, H, and I). In addition, the droplets were also easily dissolved in the presence of 1,6-hexanediol, which is suggested to disrupt hydrophobic interactions (Supplementary Fig. S5C). Changes in pH (microscopy at pH 5.5, 6.5, and 7.4) did not influence phase separation (Supplementary Fig. S5E). DLS showed a unique shift in particle size upon the addition of dsRNA (∼nm to ∼µm, Fig. 3B). Notably, the hydrodynamic radius of Ago2^IDR^ particles in the presence of dsRNA corresponds to the *in vitro* droplet size observed by microscopy (e.g. ca ∼ 2 µm, cf. Fig. 3A and B). Consistent with the microscopy data, adding ammonium acetate could reverse the particle size shift.

Next, we used 1D diffusion-ordered ^1^H NMR spectroscopy (DOSY) to characterize the translational diffusion upon condensate formation. We observed two distinct species of Ago2^IDR^ with notably different diffusion properties upon adding dsRNA (Fig. 3C). The diffusion of one state corresponds to the diffusion properties of Ago2^IDR^ alone; the other state shows a slower diffusion, potentially corresponding to the Ago2^IDR^/RNA condensate. A strong reduction of translational diffusion is consistent with observations seen for other condensates [24, 58]. Taken together, the Ago2^IDR^ shows properties of a non-specific nucleic acid binding region that establishes a dynamic coacervate.

To identify the key amino acids in the Ago2^IDR^ required for phase separation and obtain a residue-level description, we monitored the amide NMR signals in ^1^H,^15^N-HSQC spectra. Signal overlap in the central region of the spectrum reflects the disordered state of Ago2^IDR^ (Supplementary Fig. S5I), while some well-resolved signals allow more specific analyses (e.g. the side chain signals of W93 and W107). Upon adding dsRNA, significant spectral changes, both chemical shift changes and line broadening, were observed (Fig. 3D top; Supplementary Fig. S5I). The addition of sodium chloride dissolved the condensates, as seen in microscopy and turbidity assays (Supplementary Fig. S5C and H). Consistent with this, the spectral changes induced upon dsRNA binding were reversed (Fig. 3D top; Supplementary Fig. S5I), confirming an important role of electrostatic interactions for droplet formation. Since the interaction of Ago2^IDR^ with dsRNA and the droplet formation are correlated, the observed spectral changes may reflect both the partitioning in a dense droplet phase and the direct effects of the interaction of Ago2^IDR^ with dsRNA. To disentangle these contributions, the NMR sample of Ago2^IDR^ with dsRNA was centrifuged to remove condensates, and a ^1^H,^15^N-HSQC spectrum of the supernatant was compared to the phase-separated ^1^H,^15^N-HSQC spectrum (Fig. 3D bottom; Supplementary Fig. S5J). The spectrum of the supernatant mostly overlays with the spectrum of Ago2^IDR^ in the absence of dsRNA, lacking the new set of peaks. Some NMR signals remain partially shifted in the supernatant, probably due to interactions with residual RNA in the supernatant. This confirms that the observed spectral changes upon the addition of dsRNA represent the condensate state.

### The Q-rich N-terminal and repeat regions are important for Ago2^IDR^ phase separation

The Ago2^IDR^ features three regions comprising the N-terminal poly-Q region (residues 1–113, Ago2^N-term^), 11 repeats of a 23 nt long, Q-rich sequence (residues 114–365, Ago2^11-repeat^) and a C-terminal region (residues 366–413, Supplementary Fig. S4A). To test specific contributions of the different regions to RNA-binding and droplet formation, we analyzed various shortened constructs by NMR and *in vitro* phase separation assays (Fig. 3E). The ^1^H,^15^N-HSQC NMR spectra of the shorter constructs (Ago2^N-term^, Ago2^11-repeat^, and Ago2^4-repeat^) superimpose well with the corresponding signals in the full Ago2^IDR^, confirming that the Ago2^IDR^ constructs are disordered and independent of each other (Supplementary Fig. S6A and B). Fluorescence, phase contrast microscopy assays and turbidity measurements of the shorter constructs with dsRNA showed differential phase separation behavior (Fig. 3F and Supplementary Fig. S6C and D). Constructs containing one or two repeats (Ago2^1-repeat^ and Ago2^2-repeat^) showed no phase separation even at very high protein concentrations (2.5 mM and Supplementary Fig. S6D). The threshold for phase separation of Ago2^N-term^ and constructs with more repeats (Ago2^4-repeat^ and Ago2^6-repeat^) was shifted to a significantly higher concentration than Ago2^IDR^ (100 μM versus 5 μM). Ago2^IDRshort^ (comprising Ago2^N-term^, Ago2^4-repeat^, and the C-terminal region) underwent phase separation at low concentrations but to a lower extent than Ago2^IDR^ (10 μM versus 5 μM). The phase separation propensity of Ago2^11-repeat^ is only slightly reduced compared to Ago2^IDR^ (Fig. 3F and Supplementary Fig. S6C). Overall, increasing the number of repeats or combination of repeats and the Q-rich Ago2 N-terminal region enhances phase separation propensity (Fig. 3E and F).

### Ago2^N-term^ phase separation depends on charged residues

We used NMR to perform residue-specific analysis of Ago2^N-term^. Despite the highly repetitive Q-rich sequence of Ago2^N-term^ (Supplementary Fig. S7A), 65% of amide and backbone chemical shifts could be assigned. NMR secondary chemical shifts confirm the unstructured nature of the N-terminal region (Supplementary Fig. S7B). Chemical shift changes and line broadening observed in the Ago2^N-term^ dense phase (in the presence of heparin) are similar to changes seen upon the titration of Ago2^IDR^ with 21 bp hairpin dsRNA (Supplementary Fig. S7C and F). This suggests that similar interactions contribute to the phase separation of the longer Ago2^IDR^ with 21 bp hairpin dsRNA and of Ago2^N-term^ with heparin. Backbone dynamics analyzed based on NMR relaxation experiments showed significantly increased rigidity of backbone amides in the dense phase (Supplementary Fig. S7B). To assess residue-specific contributions for phase separations, we engineered two mutants, where either both tryptophan or all lysine and arginine residues are mutated to glycine. The condensate formation of Ago2^N-term^ (W/G), Ago2^N-term^ (R,K/G), and the wild type was compared by microscopy and NMR. Additionally, the effect of the different mutations was quantified in a sedimentation assay at 5°C and 25°C. While mutation of the tryptophan residues reduces phase separation slightly, mutation of the positively charged residues completely abolishes phase separation (Supplementary Fig. S7D, E, and F). These results indicate that the electrostatically driven phase separation of Ago2^N-term^ depends on interactions between positively charged residues with negatively charged moieties in heparin or nucleic acids. We note that this corresponds well to the conserved features of Ago2 N-terminal domains (Fig. 1C and Supplementary Fig. S4E).

### Ago2^4-repeat^ forms diverse protein–protein interactions in the dense phase

**Figure 4.**
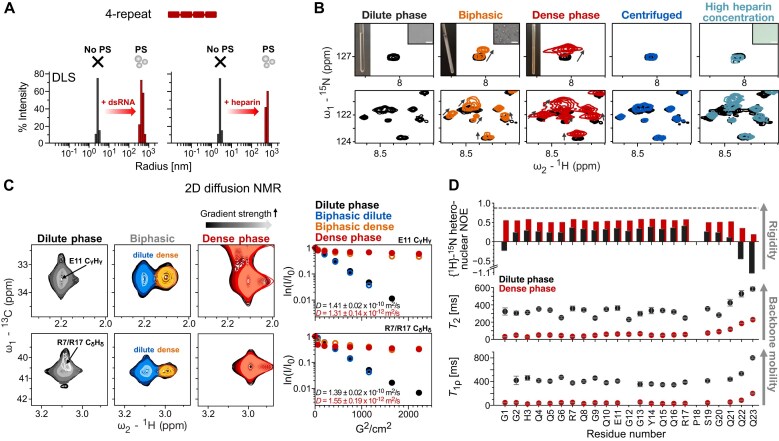
The dense phase of Ago2^4-repeat^ shows characteristic NMR chemical shifts and significantly increased rigidity. (**A**) Particle size changes upon the addition of 21 bp hairpin dsRNA or heparin to Ago2^4-repeat^ are observed by DLS. Left: Ago2^4-repeat^ (500 μM, PS-I buffer) without (black) and with 21 bp hairpin dsRNA (25 μM, red). Right: Ago2^4-repeat^ (100 μM, PS-I buffer) without (black) and with heparin (0.2 mg/ml, red). (**B**) The residual effect of phase separation was detected by NMR. Comparison of the ^1^H–^15^N HSQC spectra of Ago2^4-repeat^ (100 μM, dilute phase, black), Ago2^4-repeat^ (100 μM) with 0.2 mg/ml heparin to induce phase separation (biphasic, orange), a condensed phase sample of Ago2^4-repeat^ with heparin (∼20 mM, dense phase, red), the supernatant after centrifugation of the biphasic sample (centrifuged, blue), and after addition of 2 mg/ml heparin (high heparin concentration, light blue, PS-I buffer). (**C**) Diffusion rates of Ago2^4-repeat^ in the dilute phase (420 μM), biphase (420 μM, 1 mg/ml heparin), and dense phase (∼ 20 mM, PS-I buffer) by 2D ^1^H-^13^C NMR diffusion experiments (900 MHz ^1^H Larmor frequency). Left: Overlay of the ^1^H-^13^C NMR spectra at increasing gradient strength for residues E11 H-C_γ_ and R7/R17 H-C_δ_ for the corresponding phases. Right: Normalized intensity ratio as a function of gradient strength for E11 and R7/R17 in the corresponding phases (dilute phase: black, biphase dilute: blue, biphase dense: orange, dense phase: red). (**D**) Comparison of the backbone dynamics of Ago2^4-repeat^ in the dilute (black, 850 μM, PS-I buffer) or dense (with heparin, red) phases by ^15^N NMR relaxation experiments ({^1^H}-^15^N heteronuclear nuclear Overhauser effect (hetNOE), *T*_2_ (900 MHz ^1^H Larmor frequency), and *T*_1ρ_ (950 MHz ^1^H Larmor frequency). The error margins for the hetNOE experiment are obtained from spectral noise and the errors for *T*_2_ and *T*_1ρ_ are obtained from fitting to the exponential function.

Next, we performed further NMR experiments on the minimal Ago2 construct (Ago2^4-repeat^) that shows phase separation (Fig. 3E and F, and Supplementary Fig. S6A–C). Despite the repetitive amino acid sequence and broadening of peaks, NMR assignments allowed an amino acid-specific analysis of NMR spectra (Supplementary Figs S6A and B and S8A). Similar to Ago2^IDR^ (Fig. 3A and B), Ago2^4-repeat^ underwent protein concentration-dependent phase separation in the presence of dsRNA, with an increase in particle size correlated to dsRNA or heparin concentrations (Fig. 4A and Supplementary Fig. S8B and C).

For NMR studies, we prepared three samples representing dilute (no heparin), biphasic (addition of heparin, mixture of dilute and dense), and dense (fully phase separated) states. NMR-derived secondary structure propensity of Ago2^4-repeat^ indicates the absence of significant secondary structure for all the residues in both dilute and dense phases (Supplementary Fig. S8A). For the biphasic sample, we observed two sets of peaks in a ^1^H,^15^N-HSQC spectrum, which correspond to the NMR signals of a given spin in dilute and dense sample spectra, respectively (Fig. 4B and Supplementary Fig. S8D left). After removing the condensates by centrifugation from the biphasic NMR sample, the ^1^H,^15^N-HSQC spectrum of the supernatant represents the spectrum of the dilute phase (Fig. 4B and Supplementary Fig. S8D middle). High concentrations of heparin led to the dissolution of the condensates, as expected for the reentrant phase behavior of nucleic acid-protein condensates (Supplementary Fig. S8E). In line with this, upon adding an excess amount of heparin, we observed an NMR spectrum lacking the dense phase NMR signals (Fig. 4B and Supplementary Fig. S8D right). However, some NMR signals remain shifted compared to the dilute phase signals, suggesting that dynamic interactions of these residues with heparin could persist upon dissolution in the dilute phase (Fig. 4B and Supplementary Fig. S8D right).

We next characterized the translational diffusion of the dilute, biphasic, and dense phase samples by 2D diffusion-ordered ^1^H-^13^C NMR spectroscopy. As expected, the dense phase (in the biphasic and pure dense phase sample) showed a diffusion rate reduced by a factor of ≈ 100 compared to the dilute phase (Fig. 4C and Supplementary Fig. S8F), consistent with similar changes in diffusion reported for other condensates [58]. Moreover, NMR protein backbone ^15^N-relaxation data (*T*_1ρ_, *T*_2_, {^1^H}-^15^N heteronuclear NOE) indicate significantly reduced backbone dynamics of Ago2^4-repeat^ overall in the dense phase compared to the dilute phase, probably as a result of electrostatic protein-heparin interactions (Fig. 4D and Supplementary Fig. S8G). However, the increase of hetNOE values from the dilute (≈ 0.2) to the dense (≈ 0.5) phase does not reach the values seen in globular domains (hetNOE ≈ 0.9). This demonstrates significant residual flexibility of the polypeptide backbone in the dense phase, as has also been reported for other systems [23, 58–61].

To assess the inter/intra-molecular interactions of Ago2^4-repeat^ in the dilute and dense phases, we assessed proton-proton distances using NOESY experiments. An overlay of 2D ^1^H-^1^H NOESY spectra of Ago2^4-repeat^ dilute and dense (with heparin) phases revealed a largely increased number of inter-proton NOE cross peaks, indicating numerous contacts in the dense phase in contrast to the dilute phase (Supplementary Fig. S9A and B). While increased NOE effects are expected due to the higher concentration and reduced tumbling in the dense phase, NOE build-up curves suggest that many NOEs seen refer to direct contacts (Supplementary Fig. S9C). To our surprise, we could not observe any NMR signals corresponding to heparin despite its presence in the sample (Supplementary Fig. S9A and B). This presumably results from excessive line-broadening beyond detection due to conformational heterogeneity and dynamics at μs to ms timescales compared to the protein in the condensates.

**Figure 5.**
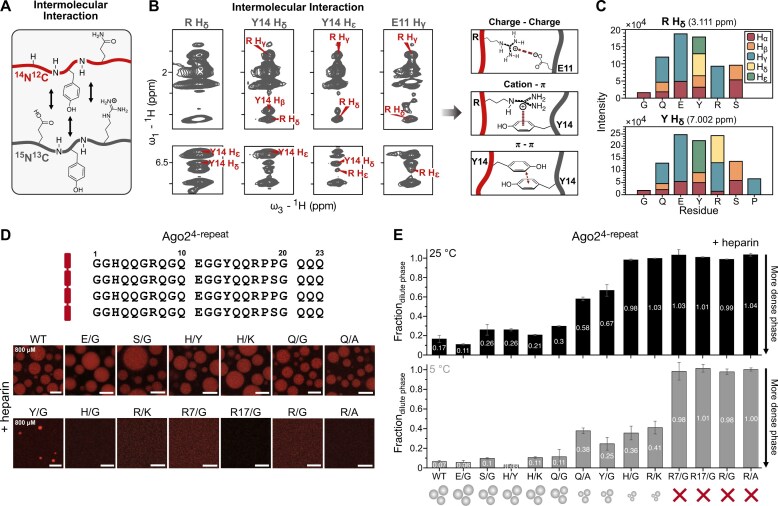
Diverse protein interactions and arginine-mediated electrostatic interactions play a role in the dense phase of Ago2^4-repeat^ and heparin. (**A**) Schematic representation of the intermolecular protein contacts visible in a filtered NOESY experiment. (**B**) Intermolecular contacts in the Ago2^4-repeat^ dense phase. Left: Strips of selected residues of the *ω*_1_–^13^C, ^15^N-filtered *ω*_3_–^15^N-edited NOESY, and *ω*_1_–^13^C, ^15^N-filtered *ω*_3_–^13^C-edited NOESY-HSQC (150 ms NOE mixing time, 1.2 GHz ^1^H Larmor frequency) of Ago2^4-repeat^ dense phase (gray). Intermolecular NOE cross peaks for which assignment was possible are labeled in red. Right: Type of interactions suggested by the intermolecular contacts to be present in the condensates. (**C**) Intensities of intermolecular NOEs (*ω*_1_–^13^C, ^15^N-filtered *ω*_3_–^13^C-edited NOESY-HSQC, mixing time 80 ms) of Arginine H_δ_ and Tyrosine H_δ_ to ^1^H, ^13^C strips show a wide range of contacts in the condensed phase. Stacked bars represent different residue types. NOE intensities are corrected for the number of residues in amino acid sequence. Only unambiguously assigned residues are reported. (**D**) Effect of amino acid composition on Ago2^4-repeat^ phase separation. Top: Sequence of Ago2^4-repeat^. All glutamates or serines or histidines or glutamines or tyrosines or arginines in the protein sequence are mutated to glycine, all histidines are mutated to tyrosines or lysines, all glutamines or arginines are mutated to alanine, all arginines are mutated to lysines or the first or second arginines in each repeat (R7 or R17) are mutated to glycines. Bottom: Fluorescent microscopy images of phase separation of Ago2^4-repeat^ WT and mutants (800 μM, PS-I buffer) with heparin (2 mg/ml) at room temperature (scale bar 10 μm). (**E**) Quantitative analysis of the effect of mutations in Ago2^4-repeat^ on condensate formation. Sedimentation assay of Ago2^4-repeat^ WT and mutants (800 μM, PS-I buffer) with heparin (2 mg/ml) at 25°C (black) and 5°C (gray). Error bars represent the standard deviation of three replicates from distinct samples.

Next, we used isotope-edited/filtered NOESY experiments to unambiguously identify intermolecular contacts in the dense phase. For this, we prepared a dense phase sample with heparin containing a mix of ^15^N,^13^C labeled and non-isotope labeled Ago2^4-repeat^ protein (ratio of 1:5) (Fig. 5A and B and Supplementary Fig. S9D–H). Analysis of the intermolecular NOEs shows, for example, contacts between arginine H_δ_ and tyrosine H_δ_/H_ϵ_ and tyrosine H_ϵ_ with arginine H_ϵ_ protons (Fig. 5B and C; Supplementary Fig. S9D and E). Arginine-tyrosine interactions might suggest the presence of cation-π interactions in the dense phase. Intermolecular NOE correlations between tyrosine H_δ_ and H_ϵ_ protons could be consistent with stacking of aromatic tyrosine rings (Fig. 5B and C; Supplementary Fig. S9D and E), while intermolecular NOEs between glutamate H_γ_ and arginine H_ϵ_ protons suggest electrostatic interactions involving arginine and glutamate side chains (Fig. 5B and C; Supplementary Fig. S9D and E). Additionally, NOE signals between arginine and glutamine side chain protons and protons of most residues present in the repeats are visible, suggesting an extensive network of contacts in the dense phase (Fig. 5B and C; Supplementary Figs S9D and E and S10A).

We observed almost identical intermolecular NOE pattern (contacts) for Ago2^4-repeat^ in the dense phase in the presence of dsDNA or heparin (Supplementary Fig. S9F). This indicates that Ago2^4-repeat^ phase separation is triggered mainly by the negatively charged backbone of dsDNA and heparin. The numerous contacts involving arginine side chains and the salt dependence of the phase separation suggest that electrostatic interactions with negatively charged sulfates in heparin or phosphates in nucleic acids also lead to close proximity of protein residues and are further stabilized by protein–-protein contacts. Taken together, the NOESY data indicate an extensive network of intermolecular contacts involving various residue types distributed across the sequence of the Ago2^IDR^.

### Arginine residues are essential for Ago2^4-repeat^ phase separation with nucleic acids

Next, to identify key contacts in the dense phase of Ago2^4-repeat^ by NMR, we introduced residue-type specific mutations in Ago2^4-repeat^, namely E/G (all Glu to Gly), S/G (all Ser to Gly), Q/G (all Gln to Gly), Q/A (all Gln to Ala), Y/G (all Tyr to Gly), H/Y (all His to Tyr), H/K (all His to Lys), H/G (all His to Gly), R/K (all Arg to Lys), R7/G (Arg at position 7 to Gly), R17/G (Arg at position 17 to Gly), R/G (all Arg to Gly), and R/A (all Arg to Ala) (Fig. 5D). Using sedimentation assays, we estimated the Ago2^4-repeat^ protein distribution between the dilute and dense phases, and turbidity assays provided information about the onset concentration for phase separation (Fig. 5D and E; Supplementary Figs S10B and C and S11A and B), also monitoring the temperature dependency of Ago2^4-repeat^ phase separation.

The mutational analysis demonstrated the pivotal role of arginines for phase separation (no condensate formation for Ago2^4-repeat^ R7/G, R17/G, R/G, or R/A), consistent with the numerous contacts observed by NMR (Fig. 5D and E; Supplementary Figs S10B and C and S11A and B). Reducing negative charges by mutating glutamic acid to glycine showed a slight enhancement of phase separation, while mutations of serine to glycine yielded only slightly reduced phase separation (Fig. 5D and E; Supplementary Figs S10B and C and S11B). Mutation of histidines to lysines or tyrosines only had a minor negative effect on phase separation in the sedimentation assay at the chosen conditions (Fig. 5D and E). However, turbidity assays show a slight shift of the onset of phase separation to lower concentrations for Ago2^4-repeat^ H/K and a significant shift of the onset of phase separation to higher concentrations for Ago2^4-repeat^ H/Y compared to wild type. Mutation of histidine to glycine significantly reduced the phase separation propensity, showing condensate formation only at lower temperatures (Fig. 5D and E; Supplementary Figs S10B and C and S11B). Ago2^4-repeat^ Q/G formed condensates at room temperature similar to Ago2^4-repeat^ WT, while Ago2^4-repeat^ Q/A, showed reduced phase separation propensity (Fig. 5D and E; Supplementary Figs S10B and C and S11B). At high protein concentrations, Ago2^4-repeat^ Y/G formed condensates at room temperature but to a lower extent than Ago2^4-repeat^ WT (Fig. 5D and E; Supplementary Figs S10B and C and S11A and B). Ago2^4-repeat^ R/K showed a similar reduction in phase separation propensity as the H/G mutation, only forming condensates at low temperatures. Removing the arginines at position 7 or 17 in all four repeats already drastically decreased the phase separation propensity, completely abolishing phase separation under conditions used for microscopy and sedimentation assays (Fig. 5D and E; Supplementary Figs S10B and C and S11A and B). Nonetheless, the addition of 2% PEG could induce phase separation for these mutants, while phase separation for R/G and R/A mutants was completely abolished in all tested conditions (Supplementary Figs S10B and C and S11B middle, C–D). These trends were confirmed by NMR measurements (Supplementary Fig. S10B and C).

While the presence of certain amino acids has a strong effect on phase separation propensity, the specific sequence and position within the repeat appear to have little effect, as shown with randomized repeats or changes in the position of arginine residues (Supplementary Fig. S11E). Based on these data, we conclude that arginine residues are essential for phase separation and likely mediate important electrostatic interactions with the negatively charged RNA.

### Coacervation of Ago2^IDR^, dsRNA, and Loqs-PD depends on Loqs-PD RNA binding

**Figure 6.**
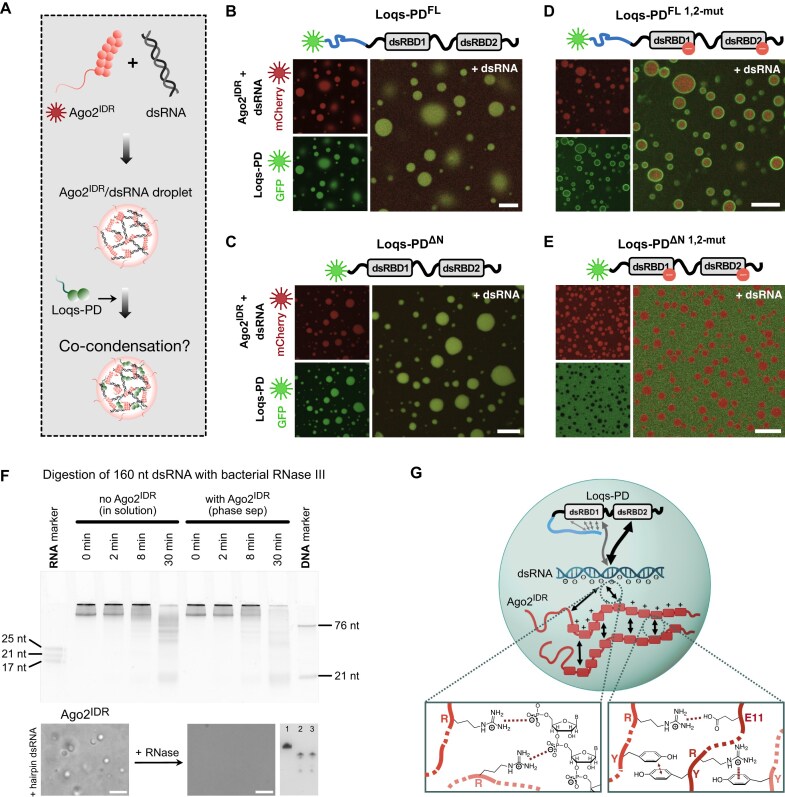
RNA binding is essential for phase separation. (**A**) Schematic overview of the experimental setup used for coacervation assays. (**B**) Coacervation of Ago2^IDR^ in the presence of 21 bp hairpin dsRNA with Loqs-PD^FL^ by fluorescent microscopy (50 μM protein, 25 μM RNA, PS-I buffer, scale bar 10 μm). (**C**) Coacervation of Ago2^IDR^ in the presence of 21 bp hairpin dsRNA with Loqs-PD^ΔN^ (N-terminal deletion mutant) by fluorescent microscopy (50 μM protein, 25 μM RNA, PS-I buffer, scale bar 10 μm). (**D**) Coacervation of Ago2^IDR^ in the presence of 21 bp hairpin dsRNA with Loqs-PD^FL 1,2-mut^ (mutant with KK to AA mutations in dsRBD1 and dsRBD2 that hinder RNA binding) by fluorescent microscopy (50 μM protein, 25 μM RNA, PS-I buffer, scale bar 10 μm). (**E**) Coacervation of Ago2^IDR^ in the presence of 21 bp hairpin dsRNA with Loqs-PD^ΔN 1,2-mut^ (mutant with N-terminal region deleted and KK to AA mutations in dsRBD1 and dsRBD2 that hinder RNA binding) by fluorescent microscopy (50 μM protein, 25 μM RNA, PS-I buffer, scale bar 10 μm). (**F**) Effect of RNase on phase separation. Top: Digestion of dsRNA upon adding bacterial RNase III (2 units of RNase III, NEB Shortcut) to the preformed Ago2^IDR^ (25 µM)/dsRNA (5 µM) coacervates. Bottom: Phase contrast microscopy images of phase separation of Ago2^IDR^ (25 μM, PS-I buffer) with 21 bp hairpin dsRNA (25 μM) in the presence and absence of RNase A (100 μg/ml, scale bar 10 μm). Cleavage after treatment with RNase A is confirmed by denaturing polyacrylamide gel (1: 25 μM 21 bp hairpin dsRNA, 2: 25 μM 21 bp hairpin dsRNA + 100 μg/ml RNase A, 3: 25 μM Ago2^IDR^ + 25 μM 21 bp hairpin dsRNA + 100 μg/ml RNase A). (**G**) Schematic model of the interactions driving the phase separation of *D. melanogaster* proteins Loqs-PD, Ago2, and dsRNA.

Since both Loqs-PD and the Ago2^IDR^ can form condensates with dsRNA, we tested whether all three components involved in siRNA biogenesis can co-partition in the same droplet, which ultimately is required during siRNA loading (Fig. 6A). GFP-Loqs-PD can enter and thus co-partition with preformed Ago2^IDR^/dsRNA droplets (Fig. 6B). To understand the contribution of different regions in Loqs-PD for the co-condensation with Ago2^IDR^ and dsRNA, we tested GFP-Loqs-PD deletion mutants (Loqs-PD^ΔC^, Loqs-PD^ΔN^, and Loqs-PD^ΔNC^) in the presence of preformed Ago2^IDR^/dsRNA droplets. The GFP-Loqs-PD deletion mutants were readily incorporated into the droplets (Fig. 6B and C; Supplementary Fig. S12A and B). To determine whether the recognition of dsRNA is required for the co-condensation, the dsRBD KK-to-AA double mutants that were analyzed in cells and *in vitro* above (see Fig. 2D and F) were examined. Neither Loqs-PD^FL 1,2-mut^ nor Loqs-PD^ΔC 1,2-mut^ mutants were able to partition into the Ago2^IDR^/dsRNA droplet phase instead, they were enriched on the surface of the Ago2^IDR^ droplets (Fig. 6D and Supplementary Fig. S12D). This suggests that the Loqs-PD mutants containing the N-terminal region form condensates with differential interfacial tensions leading to multiphase droplets [62]. When the unstructured N-terminal region of Loqs was removed (Loqs-PD^ΔN 1,2-mut^ and Loqs-PD^ΔNC 1,2-mut^) or only the N-terminal region was present (Loqs-PD^N-term^), the Loqs fusion proteins were fully excluded from the Ago2^IDR^ droplet phase and remained in the dilute phase (Fig. 6E and Supplementary Fig. S12C and E). In NMR titration experiments of Loqs-PD^FL^ and Ago2^IDR^ no direct interaction is seen (Supplementary Fig. S12G).

These results indicate that the dsRNA-binding activity of Loqs-PD is essential for co-condensation with the Ago2^IDR^/dsRNA droplets, while the N-terminal region provides additional interactions within the Ago2^IDR^/dsRNA phase. In summary, the coacervates formed by the Ago2^IDR^*in vitro* can also include Loqs, provided that its double-stranded RNA binding is not perturbed.

### The dsRNA can be cleaved by processing enzymes in the presence of condensates

We next explored whether cleavage of dsRNA, which is mediated by Dcr-2 during siRNA biogenesis, affects Ago2 condensates. To this end, we tested whether recombinant bacterial RNase III, an enzyme with RNase domains homologous to those in Dcr-2, can indeed process dsRNA in the coacervates. We observed efficient degradation of a 160 nt long dsRNA at a similar rate (Fig. 6F top, left versus right) in the absence or presence of Ago2^IDR^ (and therefore condensates), and the processed dsRNA failed to induce phase separation (Supplementary Fig. S12F). This is consistent with our observations using synthetic oligonucleotides (Supplementary Fig. S5D) and suggests that phase separation is reduced due to the decreased chain length. Similarly, when the Ago2^IDR^/dsRNA droplets were treated with RNase A, droplets dissolved readily, and the dsRNA was processed (Fig. 6F bottom). This indicates that the dsRNA is either accessible for RNA processing enzymes within the condensates or is cleaved in the dilute phase, leading to the continuous redistribution of dsRNA from the condensates and subsequent dissolution.

## Discussion

Silencing by small RNAs is an efficient process that readily culls mRNAs from the transcriptome, hence their biogenesis must be securely guided and tightly controlled to avoid unintended repression. In the miRNA processing pathway, recruitment of the target mRNA–miRNA–Ago1 complex to P-bodies is driven by GW182 condensation, facilitating the inclusion of Ago1 via multivalent interactions between GW motifs of GW182 and Ago1 [63–65]. However, in the siRNA processing pathway, *Drosophila* Ago2 does not associate with GW182 and—besides cleaving perfectly matched target mRNAs—represses translation via a distinct, GW182-independent mechanism [66]. Our data now provide evidence for the formation of dynamic coacervates involving dsRNA and the siRNA biogenesis factors Loqs-PD and Ago2 in cells and *in vitro*, which may provide a distinct environment for siRNA biogenesis. The sequence features we found important for phase separation of Ago2 are widely conserved in corresponding IDRs, indicating an important functional role for this domain and that the Ago2-dependent branch of small RNA silencing can function particularly well in the local context of a domain with a strong propensity to form condensates together with RNA.

The present study also shows that Loqs-PD forms dynamic cytoplasmic condensates in cultured cells that colocalize with Dcr-2 and Ago2 and are thus quite comparable to, yet apparently distinct from, the D2-bodies described for R2D2 (Fig. 1G and H). It is conceivable that, as a first step toward processing, the dsRBD cofactors generate condensates with suitable substrate RNAs while they are in complex with their Dicer partners, possibly to increase the efficiency and/or specificity of substrate processing. It has been proposed that Dcr-2/Loqs-PD can serve as an aRLC [11], and specific activities for Loqs-PD and R2D2 can be observed: While exogenous dsRNA and defense against acute viral infections require R2D2 [10, 67–70], the biogenesis of endo-siRNAs derived from high-copy integrated sequences depends on Loqs-PD [71, 72]. This does not exclude, however, that Dcr-2/Loqs-PD and Dcr-2/R2D2 complexes may co-occur within the same condensate.

The condensate formation of Loqs-PD depends on the RNA-binding capacity of dsRBD2, while the first dsRBD supports a liquid-like character of the condensates in cells. Although this may seem surprising given that the Loqs-PD dsRBDs have very similar structures, their differential contributions to phase separation may be affected by the presence of the linker connecting the two domains [11]. Previous studies have shown that proteins harboring multiple dsRBDs can bind to dsRNA dynamically, i.e. with independent binding of the two domains and interactions in multiple registers, allowing the protein to slide along the dsRNA [11, 73, 74]. This is consistent with the notion that multiple dsRBDs separated by linkers may function as independent, folded “stickers” that form multivalent contacts with the dsRNA ligand. The recent studies of *Drosophila* Dicer proteins together with dsRBD co-factors confirmed that in the absence of a dsRNA substrate, the dsRBDs are flexible and not observed by cryo-EM [3, 4, 75], consistent with dynamic protein-RNA interactions.

The unique intrinsically disordered N-terminal extension of Ago2 (Ago2^IDR^) harbors multiple positively charged, Q-rich repeats mediating multivalent interactions with nucleic acid binding partners. Our biophysical and NMR data, combined with mutational analyses, demonstrate that Ago2^IDR^ coacervation with dsRNA depends on electrostatic interactions involving arginine residues in the Ago2 repeats with negatively charged RNA or mimicking molecules. While the positive charge is essential for phase separation, replacement by lysine cannot fully reconstitute the phase separation behavior, as also seen in other studies [58, 76, 77]. This indicates the importance of the arginine guanidino group, which can mediate both electrostatic and π-cation interactions, thereby contributing to phase separation. Mutation of histidine to tyrosine increases the saturation concentration compared to the wild type and the mutation to lysine, suggesting a strong contribution of positive charges to phase separation. Deletion of the aromatic side chain (H/G mutant) further reduces phase separation. This also explains the stronger effect of H/G compared to Y/G mutation on phase separation.

The importance of aromatic and charged residues is further evidenced by our NOESY experiments, which indicate that arginines and aromatic residues in Ago2^IDR^ exhibit numerous intermolecular contacts, presumably through cation-π and aromatic stacking interactions in the Ago2 condensates [78]. However, the vast number of observed NOEs highlights that there is a dense network of intermolecular contacts involving many residue types. This observation and the fact that specific residue positions seem not important indicate a complex network of interactions, which is clearly distinct from a simple sticker-and-spacer model (Fig. 5B and C and Supplementary Fig. S10A). More complex intermolecular networks have also been observed for other biomolecular condensates [23, 26]. The dense interaction network also coincides with the dampened backbone dynamics of Ago2^IDR^ in the dense phase seen in the heteronuclear NOE data (Fig. 4D). A careful analysis of NOE build-ups excludes that the NOEs are dominated by spin diffusion effect but rather shows the dense network of interproton distances in the dense phase (Supplementary Fig. S9C and G), which are still observable at very short NOE mixing time (30 ms) (Supplementary Fig. S9H). A prominent feature of the Ago2^IDR^ is the presence of numerous glutamines. NOESY experiments show extensive contacts of the glutamine side chain, including glutamine–glutamine interactions. While mutation of the glutamines to glycines shows only a minor effect on condensate formation, the negative effect on phase separation is enhanced when mutating to alanine. This is consistent with a recent study on FUS, which shows that polar residues such as glycine and glutamine favor phase separation more than hydrophobic residues such as alanine [26]. The effect of glycine might be explained by an increase in flexibility that could enhance the formation of contacts between other residues.

The Ago2^IDR^/dsRNA condensates show molecular motion at a macroscopic and molecular level, indicated by the FRAP recovery, our heteronuclear NOE data, and the diffusion rates measured in the dense and dilute phases using DOSY experiments. Interestingly, in our mixed-phase sample, we observe amide signals of the dense and dilute phase at well-resolved chemical shifts in slow exchange on the NMR chemical shift time scale. Moreover, we could determine the distinct diffusion rates in the two phases for these signals, which demonstrates that the exchange between dense and dilute phase is slow on the time scale of seconds. Based on this, the estimated diffusion radius is 1.5 μm in the dense phase, consistent with the droplet sizes seen in microscopy.

The fluidity of the dense phase also allows the dsRNA to be nucleolytically cleaved by bacterial RNase III in the presence of Ago2^IDR^/dsRNA condensates. Moreover, short RNA fragments failed to induce coacervate formation with the Ago2^IDR^ (Fig. 6F and Supplementary Figs S5D and S12F). It is thus conceivable that such condensates form around long dsRNA inside cells and boost dicing into siRNAs. These will then load Ago2 and once the passenger strand is cleaved into 10 and 11 nt long sections, these short oligonucleotides will be expelled and the loaded Ago2-guideRNA complex (i.e. active RISC) is liberated. Of note, the processing of one of the essential constituents—in this case, dsRNA—within the coacervate is consistent with theoretical models for how condensates maintain their size over time rather than coalescing into ever larger structures [79].

Intriguingly, a recent publication described mutant flies with truncation of the N-terminal IDR in a mosquito Ago2 homolog (*Aedes aegypti* Ago2) that were unable to fully repress viral infection [80]. Compared to *D. melanogaster* Ago2, *Aedes aegypti* Ago2 contains a shorter (∼170 amino acids) N-terminal IDR that is nonetheless repetitive and enriched in glutamines and charged residues. These results are consistent with the hypothesis that the Ago2 N-terminal IDR augments siRNA loading efficiency in the context of a molecular condensate. It is tempting to speculate that condensation with RNA is required for efficient antiviral defense, thus explaining the particular sequence conservation pattern of the insect Ago2 N-terminal region [36].

In conclusion, our biophysical and cell biological analyses of Loqs-PD and Ago2 revealed that dynamic condensates can form together with RNA *in vitro* and *in vivo*. These require functional protein-nucleic acid interactions but also interactions among protein constituents (Fig. 6G). In addition, we identified distinct protein–protein interactions occurring within the Ago2^IDR^-nucleic acid condensate (Fig. 5B and C). This suggests that the arginine- and glutamine-rich IDRs conserved in arthropods reflect the chemical requirements for an apparently important phase separation event. For example, this could potentially enhance small RNA processing, target cleavage, or isolate the biogenesis machinery from viral inhibitors of RNA interference.

## Supplementary Material

gkaf664_Supplemental_File

## Data Availability

NMR data for assignments and analysis of dynamics are deposited in the BMRB under the accession codes 52605, 52606, and 52607. Original Western blot, gel images, and microscopy images are available in the BioStudies database (http://www.ebi.ac.uk/biostudies) under accession number S-BSST1709. Additional information is available from the lead contact upon request.
