# Supplementary material for: Molecular mechanisms of biomolecular condensate formation in Drosophila melanogaster siRNA biogenesis
Source: Nucleic Acids Res. 2025 Jul 19;53(14):gkaf664. doi: 10.1093/nar/gkaf664 (PMC12276012; doi:10.1093/nar/gkaf664)
Supplement: gkaf664_Supplemental_File [file gkaf664_supplemental_file.pdf]

Supplementary Information

Molecular mechanisms of biomolecular condensate formation in *Drosophila melanogaster* siRNA biogenesis

Clara Hipp[1,2], Selina Mussgnug[3], Purva Choudhary[3], Hyun-Seo Kang[1,2], Sam Asami[1,2], Judit Sastre[4], Carsten Donau[4], Romy Böttcher[3], Gerd Gemmecker[1,2], Job Boekhoven[4], Klaus Förstemann[3,*], Michael Sattler[1,2,*]

* To whom correspondence should be addressed. Email: michael.sattler@helmholtz-munich.de or foerstemann@genzentrum.lmu.de

Contents

Supplementary Results **2**

Supplementary Methods **6**

Supplementary Figures **9**

Supplementary Tables **31**

Supplementary References **35**

SUPPLEMENTARY RESULTS

Construct-dependent phase separation of Loqs-PD

We generated several deletion mutants of Loqs-PD with either the N-terminal region (Loqs-PD$^{\Delta N}$, the C-terminal region (Loqs-PD$^{\Delta C}$), or both regions (Loqs-PD$^{\Delta NC}$) deleted, and a construct that only contains the unstructured Loqs-PD N-terminal region (Loqs-PD$^{N\text{-}term}$). While deletion of the C-terminal region only shows a small effect on the phase separation behavior, deletion of the N-terminal intrinsically disordered region significantly reduces droplet formation (Loqs-PD^{FL} and Loqs-PD$^{\Delta C}$ when compared to the Loqs-PD$^{\Delta N}$ and Loqs-PD$^{\Delta NC}$) although Loqs-PD$^{N\text{-}term}$ does not phase separate on its own at the conditions tested. The addition of 1 % PEG to mimic the crowded environment in the cell further enhanced droplet formation for all Loqs-PD constructs containing the dsRBDs (**Figure 2C**; **Supplementary Figure S2G**). Increasing the concentration of PEG to 5% already induces phase separation at low protein concentration (10 μM) and physiological salt concentration (150 mM salt) (**Supplementary Figure S2G**).

Intramolecular interaction of Loqs-PD N-terminal region with dsRBD1

To test the presence of potential intramolecular interactions we compared the NMR spectra of the four deletion mutants of the Loqs-PD^{FL} (**Figure 2B**; **Supplementary Figure S2A**) The NMR spectra of dsRBD12-containing constructs (Loqs-PD^{FL}, Loqs-PD$^{\Delta C}$, Loqs-PD$^{\Delta N}$, Loqs-PD$^{\Delta NC}$) are overall very similar and can be superimposed for the common regions, with well-dispersed signals corresponding to the folded dsRBD1 and dsRBD2 domains and signals in the crowded central region corresponding to unstructured N- and C-terminal regions (**Supplementary Figure S2A**). Chemical shift differences are observed for amides in dsRBD1 when comparing the ^{1}H, ^{15}N-HSQC spectra of Loqs-PD$^{\Delta NC}$ and Loqs-PD$^{\Delta C}$ (**Figure 2B**). Similarly, the end of the N-terminal region of Loqs-PD (residue 100-125) shows chemical shift changes when comparing ^{1}H, ^{15}N-HSQC spectra of Loqs-PD$^{\Delta C}$ Loqs-PD$^{N\text{-}term}$ (**Supplementary Figure S2B** top). This suggests that the residues at the end of the Loqs-PD N-terminal region intramolecularly interact with residues in the dsRBD1.

Salt-dependency of RNA-induced phase

NMR spectroscopy is used to identify the residues and regions in Loqs-PD that are affected by RNA-induced phase separation. For this, we performed NMR titrations of Loqs-PD$^{\Delta C}$ with 21 bp hairpin dsRNA at three salt concentrations (50 mM, 150 mM, and 500 mM, respectively). These

experiments show that phase separation diminishes gradually with increasing ionic strength of the buffer (**Supplementary Figure S2H**). In all the conditions, NMR signals corresponding to dsRBD1 and dsRBD2 were severely broadened upon the addition of dsRNA. However, for the conditions where phase separation occurs (lowest and physiological salt concentration, 50 mM and 150 mM, respectively), line-broadening is also observed for amides in the N-terminal region and the linker connecting the two dsRBDs upon adding dsRNA. At the highest salt concentration, 500 mM, where no phase separation is observed, no line-broadening is evident for these regions.

The structural integrity of the dsRBD mutants

To ensure that the mutants with KK to AA mutations in dsRBD1 and 2 maintain the overall structure of the dsRBDs, the $^{1}H,^{15}N$-HSQC spectrum of Loqs-PD$^{\Delta C}$ is compared to the $^{1}H,^{15}N$-HSQC spectra of Loqs-PD$^{\Delta C\ 1\text{-}mut}$ and Loqs-PD$^{\Delta C\ 2\text{-}mut}$, respectively. Chemical shift differences comparing wildtype and mutant dsRBDs map to residues that are in close spatial proximity to the mutation sites (**Supplementary Figure S3A**). This confirms that the mutations do not affect the overall fold of the dsRBDs and mostly impair contacts with RNA.

Differential effect of dsRBD1 and dsRBD2 mutation on phase separation

Turbidity assays confirmed that Loqs-PD$^{\Delta C\ 2\text{-}mut}$ does not form condensates in the presence of dsRNA and showed a slightly higher phase separation propensity for Loqs-PD$^{\Delta C\ 1\text{-}mut}$ than Loqs-PD$^{\Delta C}$ wildtype (**Supplementary Figure S3E** top). In line with this, a comparison of NMR spectra of Loqs-PD$^{\Delta C}$ mutants showed less or no line broadening in the N-terminal and linker regions for Loqs-PD$^{\Delta C\ 2\text{-}mut}$ and Loqs-PD$^{\Delta C\ 1,2\text{-}mut}$, while Loqs-PD$^{\Delta C\ 1\text{-}mut}$ showed increased line broadening compared to Loqs-PD$^{\Delta C}$ (**Supplementary Figure S3C**).

The intramolecular interaction of the N-terminal region of Loqs-PD and dsRBD1 is not affected by the dsRBD mutations

Generally, all constructs of Loqs-PD lacking the N-terminal region (Loqs-PD$^{\Delta NC}$, Loqs-PD$^{\Delta NC\ 1\text{-}mut}$ and Loqs-PD$^{\Delta NC\ 2\text{-}mut}$) show a lower phase separation tendency. Comparison of turbidity assays and NMR titrations of these mutants lacking the N-terminal region (Loqs-PD$^{\Delta NC}$, Loqs-PD$^{\Delta NC\ 1\text{-}mut}$ and Loqs-PD$^{\Delta NC\ 2\text{-}mut}$) with 21 bp dsRNA show similar tendencies in the phase separation behavior as the constructs that include the N-terminal region (with dsRBD1 or dsRBD2 mutated showing slightly enhanced or no phase separation, respectively,

Supplementary Figure S3C, E). The spectral signatures that are indicative of this intramolecular interaction are still seen in the dsRBD1 mutant (comparing Loqs-PD$^{\Delta NC\ 1\text{-}mut}$ vs Loqs-PD$^{\Delta C\ 1\text{-}mut}$, **Supplementary Figure S3F**), suggesting that the dsRBD1 KK to AA mutation does not affect the intramolecular interaction. This confirms that the intramolecular interactions of the N-terminal region with dsRBD1 and the differences in the behavior of Loqs-PD mutants (Loqs-PD$^{\Delta NC\ 1\text{-}mut}$ and Loqs-PD$^{\Delta NC\ 2\text{-}mut}$) are not connected.

Mutation of aromatic and charged residue in Ago2$^{N\text{-}term}$

The side chain NMR signals of two tryptophan residues present in Ago2$^{N\text{-}term}$ are shifted upon phase separation in ^{1}H,^{15}N-HSQC spectra (**Supplementary Figure S7C**). Moreover, there are 11 lysines and 3 arginines in the Ago2$^{N\text{-}term}$ sequence (**Supplementary Figure S7A**). Since aromatic and charged residues are known as drivers of phase separation and the tryptophan residues are affected by the induction of phase separation in the NMR spectra, we engineered two mutants, where either both tryptophan or all lysine and arginine residues are mutated to glycines.

Mutation of aromatic and charged residue in Ago2$^{4\text{-}repeat}$

We recorded NMR spectra for all mutants at conditions where they form droplets. As for Ago2$^{4\text{-}repeat}$ WT, we observed two sets of signals in ^{1}H,^{15}N-HSQC NMR spectra recorded at 25 °C for Ago2$^{4\text{-}repeat}$ Q/G, (100 μM), Ago2$^{4\text{-}repeat}$ E/G, Ago2$^{4\text{-}repeat}$ S/G, Ago2$^{4\text{-}repeat}$ Q/A, Ago2$^{4\text{-}repeat}$ H/Y and Ago2$^{4\text{-}repeat}$ H/K corresponding to the dilute and dense phases. For Ago2$^{4\text{-}repeat}$ Y/G, H/G, and R/K (800 μM) samples were recorded at 5 °C to achieve phase separation, while Ago2$^{4\text{-}repeat}$ R7/G and R17G required an addition of 2% PEG to induce phase separation. No phase separation was detected for Ago2$^{4\text{-}repeat}$ R/G and R/A, even at low temperatures and in the presence of 2% PEG as a crowding reagent (**Supplementary Figure S10B, C**). A turbidity assay at 25 °C and 5 °C and increasing protein concentrations confirm the observed tendencies (**Supplementary Figure S11B**). DLS of Ago2$^{4\text{-}repeat}$ Y/G, H/G, R/K, and R/G (100 μM) before and after the addition of heparin at 25 °C and cooled displayed an increase in particle size, indicating phase separation, for Ago2$^{4\text{-}repeat}$ H/G, Y/G, and R/K but not for R/G upon cooling. This confirms that the phase separation of Ago2$^{4\text{-}repeat}$ H/G, Y/G, and R/K is temperature-dependent and that the R/G mutation abolishes phase separation (**Supplementary Figure S11A**).

Digestion of dsRNA with RNase III

To analyze if the processing of dsRNA is possible in the presence of Ago2^{IDR}/dsRNA condensates, a digestion assay was performed (**Figure 6F; Supplementary Figure S12F**). Since *in vitro* experiments with Dcr-2 are difficult to perform, we simplified this scenario with recombinant bacterial RNase III, an enzyme that is significantly smaller but homologous to the Dcr-2 RNase domains and also contains a dsRBD. The enzyme forms an intermolecular dimer (while Dcr-2 bears tandem RNase III-domains); hence, it will also cleave both strands of dsRNA. To assess whether the cleavage of the dsRNA affects the coacervate formation with the Ago2^{IDR}, the dsRNA was pre-digested at different times with bacterial RNase III before mixing with the Ago2^{IDR}. The fully processed dsRNA length will be roughly 11 nt of dsRNA when using bacterial RNase III (see also **Figure 6F**). After 10 minutes of digestion, the dsRNA concentration required to induce a substantial increase in turbidity, i.e., coacervate formation, was shifted from 2.5 to 5 µM, while after 30 minutes of digestion, even the highest concentration tested (10 µM) did not result in coacervate formation (**Supplementary Figure S12F**).

SUPPLEMENTARY METHODS

Cloning of Loqs-PD constructs for experiments in cells

The muGFP-Loqs-PD variants were assembled from independent building blocks for the N-terminus, dsRBD-containing core and C-terminus. The fragments were ordered as individual gBlocks (IDT) and assembled in a modular fashion. The gBlock coding for the monomeric GFP version muGFP + in-frame *Kpn*I-site was inserted *Bam*HI-*Not*I into pKF254(1) downstream of the ubiquitin promoter yielding plasmid pLT2. Separately, the Loqs-PD core (or mutants thereof) with a silent *Aat*I-site engineered at the junction with the N-terminal part was inserted *Kpn*I-*Not*I into a pUC19 plasmid with a deleted *Aat*I site in the polylinker. Wild-type or mutant N-terminal fragments were then added *Kpn*I-*Aat*I and the resulting fragment was joined with muGFP by transferring it *Kpn*I-*Not*I into pLT2.

Estimation of the cytoplasmic concentration of Loqs-PD

For the determination of the cytoplasmic Loqs-PD concentration, a volume of approximately 523 μm^3 for one cell and a volume of approximately 67 μm^3 for the nucleus were measured from microscopy images, resulting in a volume of approximately 456 μm^3 for the cytoplasm ($V_{cytoplasm}$). The protein was extracted from 20000 S2 cells (N_{cells}), and 100 fmol of purified protein ($c_{purified}$) was used for the western plot as a comparison. The cytoplasmic concentration is calculated from the band intensity ratio of protein extracted from S2 cells to purified protein ($I_{cells/purified}$) as shown below:

$$C_{cytoplasmic} = \frac{c_{purified} \times I_{cells/purified}}{V_{cytoplasm} \times N_{cells}}$$

The calculated values for the four replicates are shown in **Table S2**. The values are estimated, and the standard deviation is calculated.

Estimation of the protein concentration within the dense phase sample of $Ago2^{4-repeat}$/heparin and the dsDNA concentration within the dense phase sample of $Ago2^{4-repeat}$/21 bp dsDNA

To estimate the concentration of $Ago2^{4-repeat}$ within the $Ago2^{4-repeat}$/heparin condensates, the dense phase sample was prepared as described in the methods. 1 µl of the dense phase was diluted in buffer (1:10, 20 mM Phos, pH 6.5, 500 mM NaCl) and the absorption at 280 nm was measured. Due to the high viscosity of the dense phase, the value only results in a rough estimation of the concentration. This yielded an $Ago2^{4-repeat}$ concentration within the dense phase of approximately 20 mM.

Similarly, the concentration of 21 bp dsDNA within the $Ago2^{4-repeat}$/21bp dsDNA condensates was estimated. 1 µl of the dense phase was diluted in buffer (1:100, 20 mM Phos, pH 6.5, 500 mM NaCl) and the absorption at 260 nm was measured. Due to DNA's higher mass attenuation coefficient at 260 nm compared to protein, the DNA concentration can be estimated although a high protein concentration of $Ago2^{4-repeat}$ is present in the dense phase. This yielded a DNA concentration within the dense phase of approximately 35 mM ($A_{260/280}$ = 1.6).

To confirm the presence of heparin in the condensed phase (which is not possible based on extinction), we used a 2D $^1H,^1H$ NOESY experiment. We formed $Ago2^{4-repeat}$/heparin dense phase and diluted 10 µl dense phase in 150 µl buffer with high salt concentration to dissolve the phase separation (20 mM sodium phosphate, pH 6.5, final salt concentration: 500 mM NaCl). We then performed 2D $^1H,^1H$ NOESY experiments on this sample and compared it to a 2D $^1H,^1H$ NOESY experiment of heparin (20 mg/ml) in the same buffer.

Production of T7 RNA polymerase

N-terminally His_6-tagged T7 RNA polymerase (pQE vector) is expressed in *E. coli* M15 (pREP4) cells. The cells were grown in LB medium at 37 °C to an OD_{600} of 0.6 and subsequently induced with 0.2 mM IPTG. The T7 RNA polymerase was expressed at 20 °C overnight. The cells were harvested (7808 × *g*) and resuspended in 50 mM Tris-HCl, pH 8.0, 300 mM NaCl, 10 mM $MgSO_4$, 10 µg/ml DNase I, 1 mM AEBSF.HCl, 1 mM β-mercaptoethanol, 1 mg/ml lysozyme. After lysis by sonication (2 x 3 min on ice-water) and centrifugation (38759 × *g*, 1 h), the supernatant is filtered over a 0.22 µm filter, and imidazole is added to a final concentration of 20 mM. The supernatant is loaded on a 5 ml HiTrap chelating column charged with $NiCl_2$ (equilibrated with

Ni binding buffer: 50 mM Tris-HCl, pH 8.0, 300 mM NaCl, 20 mM imidazole and 1 mM β-mercaptoethanol), washed with Ni binding buffer containing 50 mM imidazole and eluted (Ni elution buffer: 50 mM Tris-HCl, pH 8.0, 300 mM NaCl, 300 mM imidazole and 1 mM β-mercaptoethanol). The protein fractions are dialyzed at 4°C overnight against HyAp-binding buffer (20 potassium phosphate, pH 7.5, 50 mM KCl, 0.05 mM EDTA, 3 mM β-mercaptoethanol). The protein is applied to a Bio-Gel HT Hydroxyapatite column (BioRad) equilibrated with HyAp-binding buffer and eluted with HyAp-elution buffer (20 potassium phosphate, pH 7.5, 50 mM KCl, 0.05 mM EDTA, 3 mM β-mercaptoethanol, 568 mM ammonium sulfate). The protein fractions are pooled, dialyzed to storage buffer (20 potassium phosphate, pH 7.5, 100 mM NaCl, 1 mM EDTA, 1 mM DTT, 50% (v/v) glycerol) and stored at a protein concentration of 10 mg/mL at -80 °C.

SUPPLEMENTARY FIGURES

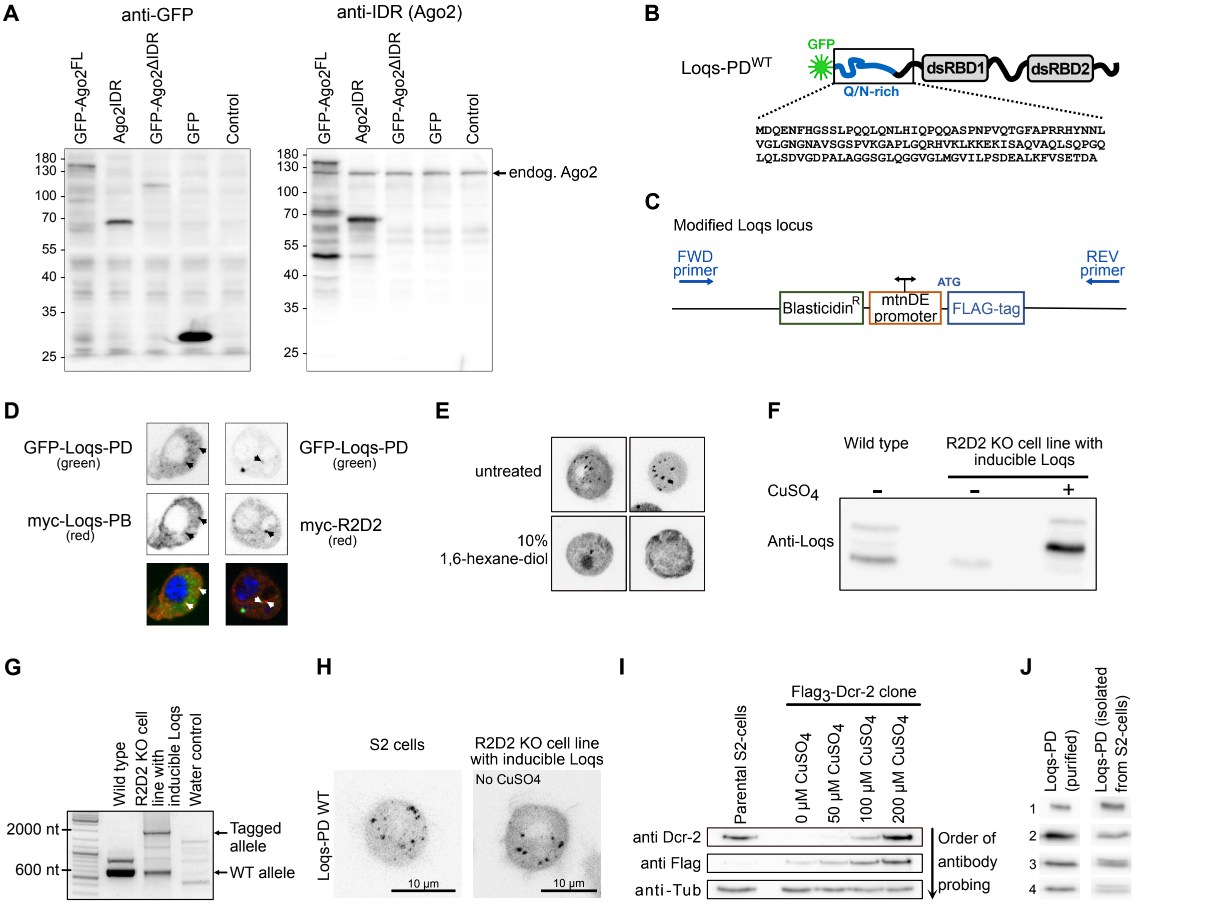

Figure S1. Characterization of Loqs-PD dsRBD mutants and R2D2 KO cell line with inducible Loqs. (A) Western Blot analysis of the expression levels for the GFP-Ago2 fusion proteins in our stable cell lines. (B) Domain structure of Loqs-PD. The sequence of the N-terminal Q/N rich region is shown below. (C) Schematic drawing of modified *loqs* locus upon genome editing. The position of primers used for PCR of genomic DNA in e) is indicated by blue arrows. (D) Co-staining of GFP-Loqs-PD with myc-Loqs-PB (left) and myc-R2D2 (right). (E) Treatment of S2 cells with 10% 1,6-hexanediol for 1 h leads to the disappearance of spots, arguing that the condensation is reversible. (F) Western blot (anti-Loqs) of wild-type cells and the R2D2 KO cell line with inducible Loqs with (10 µM $CuSO_4$) or without Loqs induction. Note that this cell line still seems to contain at least one unmodified *loqs* allele. (G) PCR of genomic DNA isolated from wild-type cells, R2D2 KO cell line with inducible Loqs (and water control without DNA) on a 1% agarose gel. (H) S2 cells (wildtype) transfected with Loqs-PD (left, same cell as shown in **Figure 1G**) and R2D2 KO cell line with inducible Loqs, uninduced (right). (I) The inducible *mtnDE*-Promoter $Flag_3$-Dcr-2 cell line shows robust induction upon the addition of $CuSO_4$ with a very slight "leaky" expression of $Flag_3$-Dcr2 in the uninduced state. (J) Estimation of cytoplasmic Loqs-PD concentration. Loqs-PD is isolated from S2 cells (20.000) and the band intensity in a western blot is compared to purified protein to obtain a cytoplasmic protein concentration of 10.5 ± 5.6 µM. The experiments were performed in 4 replicates, and the mean and standard deviation were calculated. The bands for each replicate were cropped from the same exposure + grayscale settings to show them in this figure.

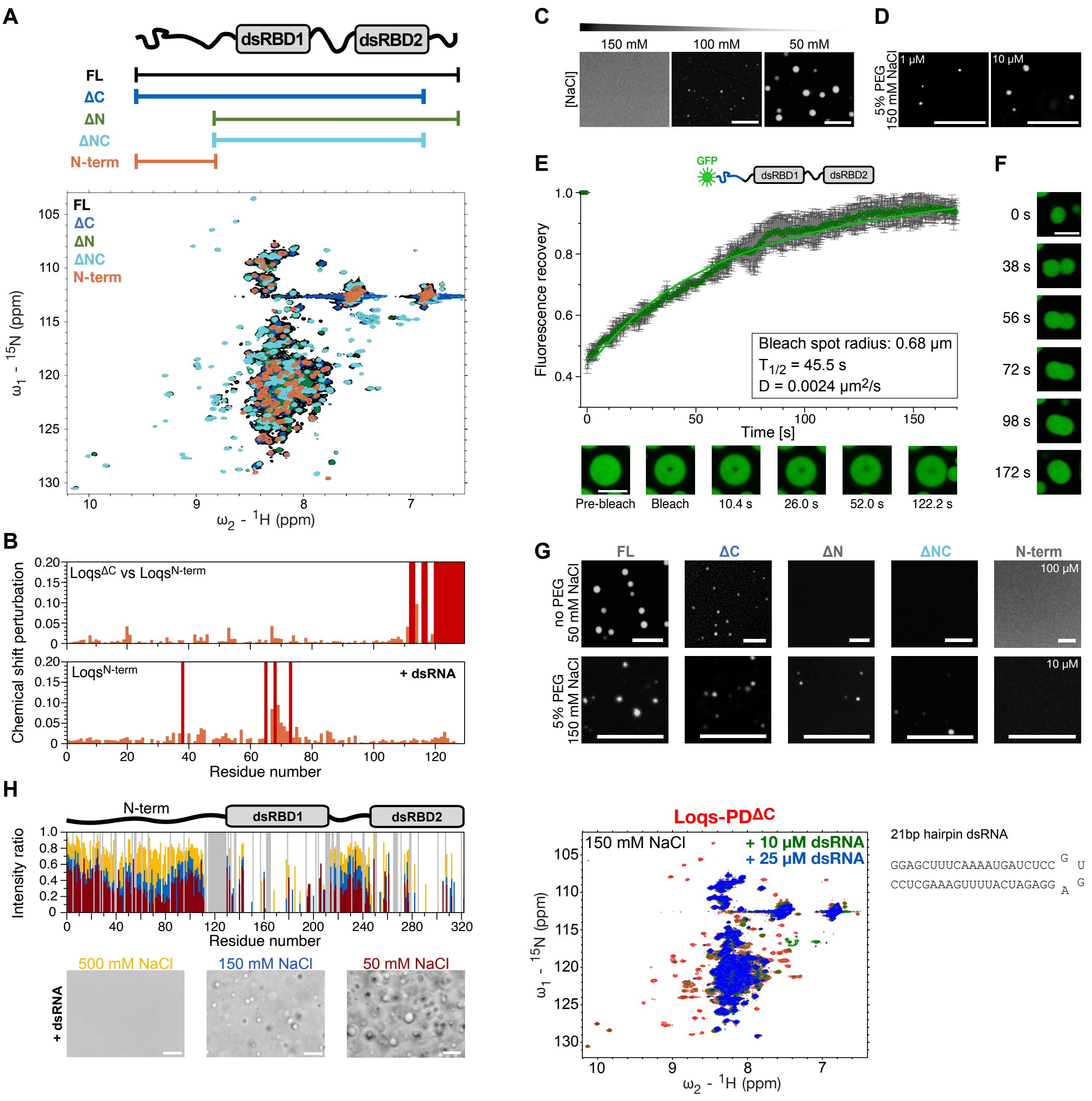

Figure S2. Phase separation properties of Loqs-PD. (A) Overlay of the ^{1}H–^{15}N HSQC spectra of Loqs-PD FL (black), Loqs-PD$^{\Delta C}$ (blue), Loqs-PD$^{\Delta N}$ (green), Loqs-PD$^{\Delta NC}$ (cyan), and Loqs-PD$^{N\text{-}term}$ (orange) (100 μM, PS-II buffer, 292 K) (related to **Figure 2B**). (B) Top: CSP of the comparison of ^{1}H–^{15}N HSQC of Loqs-PD$^{\Delta C}$ and Loqs-PD$^{N\text{-}term}$ shows chemical shift changes between residues 110-129. Bottom: CSP of the ^{1}H–^{15}N HSQC titration of Loqs-PD$^{N\text{-}term}$ (100 μM, PS-I buffer) with 21 bp hairpin dsRNA (25 μM RNA) shows RNA binding. Residues that are disappearing are set to a value of 0.2 and colored red. (C) Salt-dependent phase separation of Loqs-PD (100 μM, 20 mM sodium phosphate, pH 6.5) by fluorescent microscopy. (D) Fluorescent microscopy images of Loqs-PD^{FL} at low concentrations (1 and 10 μM, PS-II buffer, 5% PEG8000, scale bar 10 μm). (E) Slow fluorescence recovery of Loqs-PD$^{\Delta C}$ in the presence of 21 bp hairpin dsRNA in FRAP experiments (50 μM protein, 25 μM RNA, PS-II buffer, bleach spot radius ≈ 0.7 μm, droplet radius ≈ 5.24 μm, scale bar 10 μm). Top: fluorescence recovery curve over time. The error is calculated from 3 replicates of FRAP measurements of the same sample. Bottom: fluorescence microscopy images of one replicate at selected time points during the FRAP experiment. (F) Fusion of two droplets of Loqs-PD$^{\Delta C}$ in the presence of 21 bp hairpin dsRNA (50 μM protein, 25 μM RNA, PS-II buffer). (G) Loqs-PD dsRBDs are essential for phase separation. Fluorescent microscopy images of Loqs-PD constructs at high protein concentrations and low salt concentrations (100 μM protein concentration, PS-I buffer, scale bar 10 μm) and at lower protein and physiological salt

concentration with 5% PEG (10 μM protein concentration, PS-II buffer) (related to **Figure 2C**). (H) The RNA-induced phase separation of Loqs-PD is salt-dependent. Top left: comparison of the intensity ratio of NMR titrations of Loqs-PD$^{\Delta C}$ (100 μM, 20 mM sodium phosphate, pH 6.5, 292 K) with 21 bp hairpin dsRNA (25 μM) at various salt concentrations (yellow: 500 mM NaCl, blue: 150 mM NaCl, red: 50 mM NaCl). Bottom left: phase contrast microscopy images of Loqs-PD$^{\Delta C}$ (100 μM, 20 mM sodium phosphate, pH 6.5) with 21 bp hairpin dsRNA (25 μM) at various salt concentrations (Scale bar 10 μm). Right: NMR titration of Loqs-PD$^{\Delta C}$ (100 μM, PS-II buffer, 292 K) with 21 bp hairpin dsRNA (up to a molar ratio of 1:0.25.

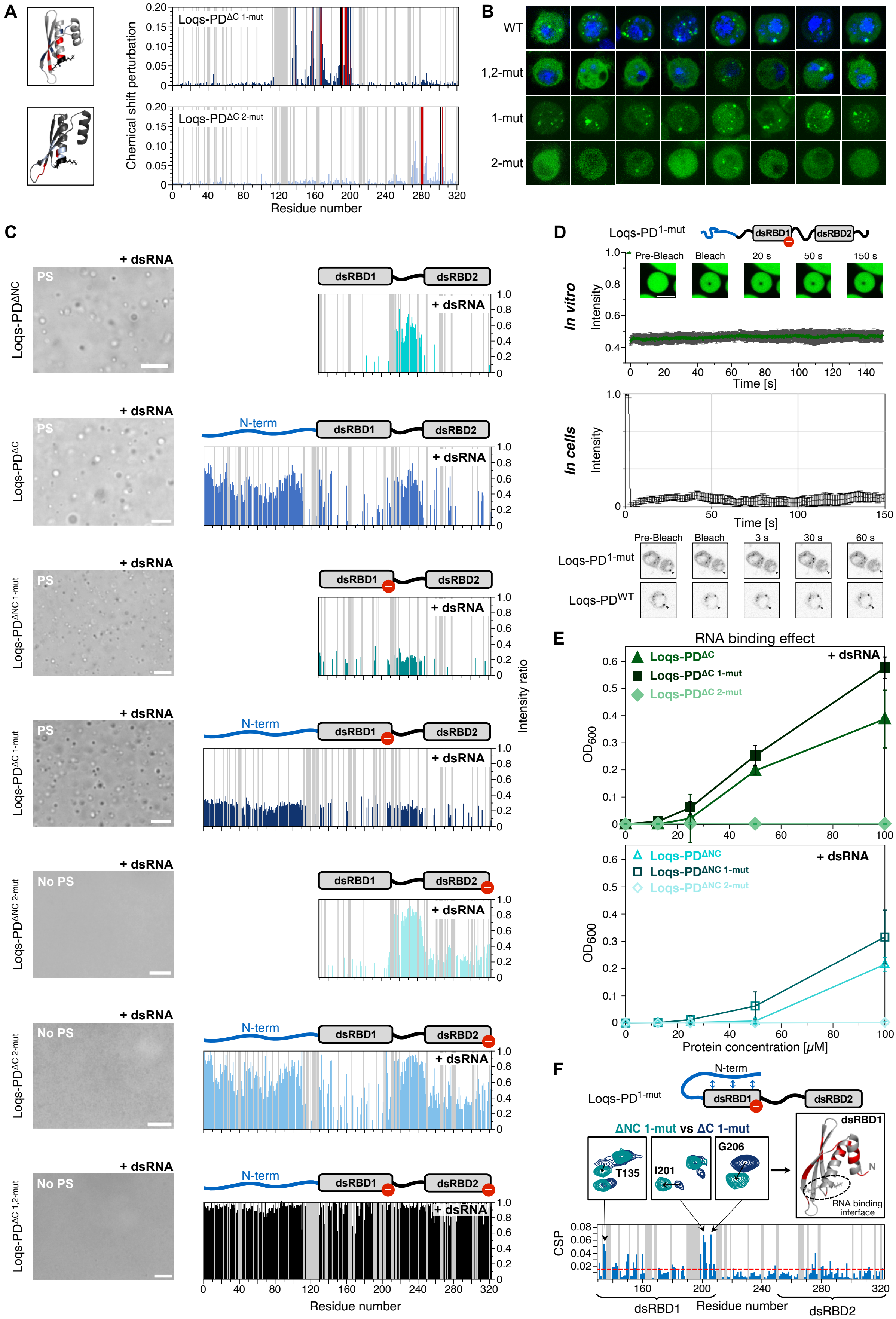

Figure S3. Effect of mutations of the RBDs on the phase separation of Loqs-PD. (A) CSP of the comparison of ^{1}H–^{15}N HSQC of Loqs-PD$^{\Delta C}$ and Loqs-PD$^{\Delta C\ 1\text{-mut}}$ (top) and Loqs-PD$^{\Delta C}$ and Loqs-PD$^{\Delta C\ 2\text{-mut}}$

(bottom) shows chemical shift changes of residues close to the KK to AA mutations (PS-II buffer). In Loqs-PD$^{\Delta C\ 1\text{-mut}}$ and Loqs-PD$^{\Delta C\ 2\text{-mut}}$, dsRBD1 or dsRBD2 are mutated (K189, K190 for Loqs-PD$^{\Delta C\ 1\text{-mut}}$ and K301, K302 for Loqs-PD$^{\Delta C\ 2\text{-mut}}$) to suppress RNA binding to the respective dsRBD. KK to AA mutations are shown in black and residues that are disappearing are set to a value of 0.2 and colored red. CSP > 0.03 are marked on the dsRBD1 structure (pdb: 5NPG) in dark blue and the dsRBD2 structure in light blue (pdb: 5NPA). Residues that are disappearing are colored red and the mutated lysine residues are shown as sticks in black. (B) Fluorescent microscopy images of different Loqs-PD constructs in cells (related to **Figure 2F**). (C) Mutational effect on phase separation behavior of Loqs-PD. Left: droplet formation of Loqs-PD$^{\Delta NC}$, Loqs-PD$^{\Delta C}$, Loqs-PD$^{\Delta NC\ 1\text{-mut}}$, Loqs-PD$^{\Delta C\ 1\text{-mut}}$, Loqs-PD$^{\Delta NC\ 2\text{-mut}}$, Loqs-PD$^{\Delta C\ 2\text{-mut}}$ and Loqs-PD$^{\Delta C\ 1,2\text{-mut}}$ in the presence of 21 bp hairpin dsRNA (75 μM protein, PS-II buffer, scale bar 10 μm). Right: intensity changes of the NMR titration of these Loqs-PD constructs (100 μM, PS-II buffer, 292 K) with 21 bp hairpin dsRNA (25 μM). Not assigned peaks are shown in grey. (D) No fluorescence recovery of Loqs-PD$^{1\text{-mut}}$ *in vitro* and in cells. Top: FRAP experiments (50 μM protein, 25 μM RNA, PS-II buffer, bleach spot radius ≈ 0.8 μm, droplet radius ≈ 5.6 μm, scale bar 10 μm). The error is calculated from 3 replicates of FRAP measurements of the same sample. Bottom: FRAP experiments of GFP-Loqs-PD$^{1\text{-mut}}$ spots (± SE, n=17) in S2-cells demonstrate no fluorescence recovery. The fluorescence recovery at selected time points is shown in comparison to Loqs-PD^{WT} below. (E) The effect of the dsRBD mutation on droplet formation is independent of the Loqs-PD N-terminus. Top: turbidity assay (OD_{600}) of Loqs-PD$^{\Delta C}$ (triangle, green), Loqs-PD$^{\Delta C\ 1\text{-mut}}$ (square, dark green) and Loqs-PD$^{\Delta C\ 2\text{-mut}}$ (diamond, light green) in the presence of 21 bp hairpin dsRNA (25 μM RNA, PS-II buffer). Bottom: turbidity assay (OD_{600}) of Loqs-PD$^{\Delta NC}$ (triangle, cyan), Loqs-PD$^{\Delta NC\ 1\text{-mut}}$ (square, petrol blue) and Loqs-PD$^{\Delta NC\ 2\text{-mut}}$ (diamond, light blue) in the presence of 21 bp hairpin dsRNA (25 μM RNA, PS-II buffer). Error bars represent the standard deviation of three replicates from distinct samples. (F) Intramolecular interaction of the N-terminal region of Loqs-PD and dsRBD1 is not affected by dsRBD1 mutation. Exemplary regions of shifted peaks in the ^{1}H–^{15}N HSQC comparison of Loqs-PD$^{\Delta NC\ 1\text{-mut}}$ (turquoise) and Loqs-PD$^{\Delta C\ 1\text{-mut}}$ (dark blue) are shown, and chemical shift perturbation (CSP) is plotted against residue number in blue. CSP > 0.015 are marked on the dsRBD1 structure (pdb: 5NPG) in red, RNA binding site is highlighted, and key lysine residues are shown as sticks.

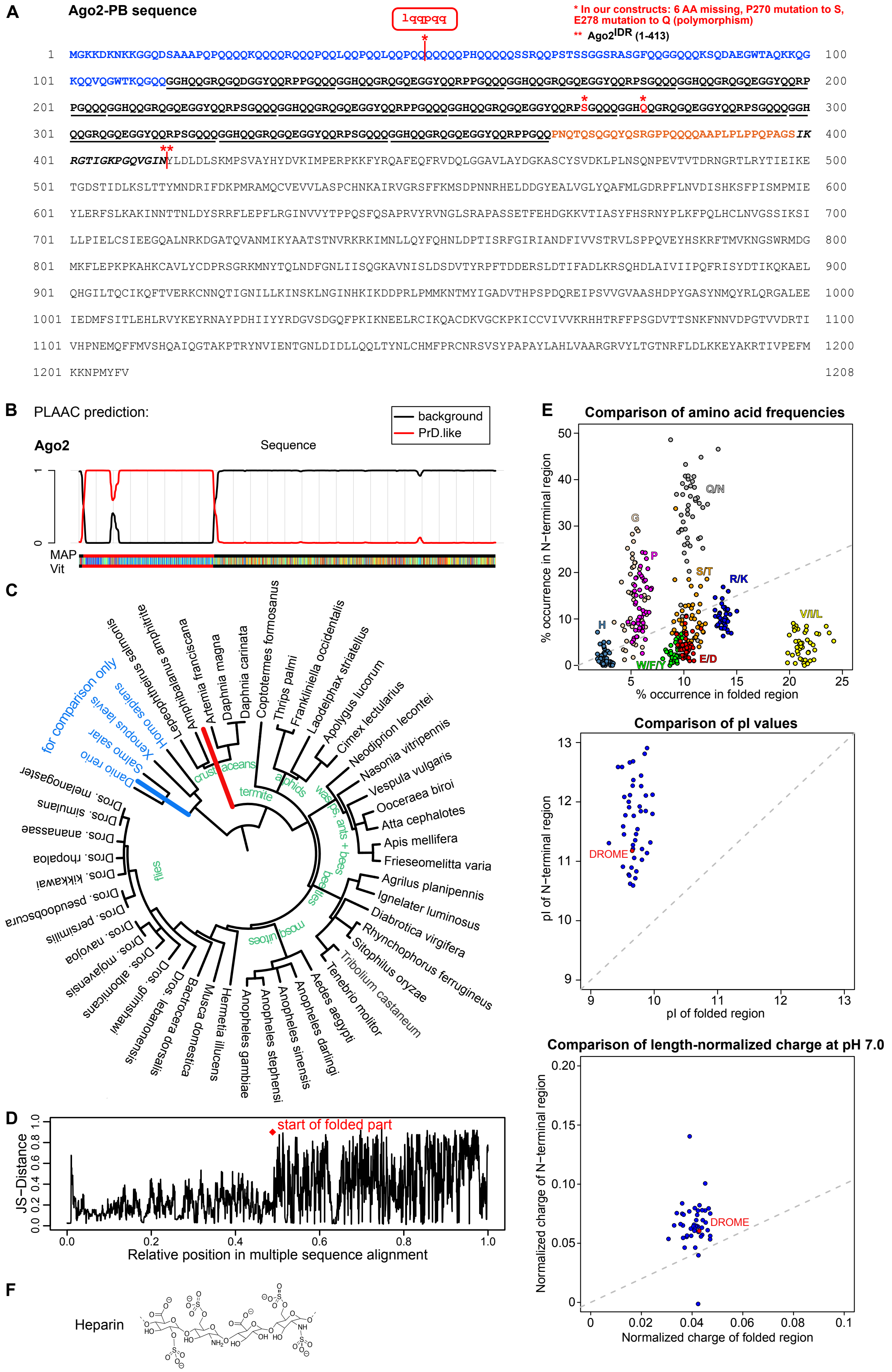

Figure S4. Ago2 sequence properties and PLAAC prediction. (A) Sequence of the Ago2-PB construct used in our experiments. Compared to the FlyBase sequence, the sequence has a 6 amino acid deletion, a P270 to S mutation, and an E278 to Q mutation (polymorphism, marked by red * in sequence; indexing

numbers are based on our construct). Ago2^{IDR} contains amino acids 1-413 (polyQ-containing sequence 1-113 (blue), repetitive sequence 114-365 (repeat: 23 AA, bold black, underlined), and connector sequence 366-398 (orange). (B) Prion-Like Amino Acid Composition (PLAAC) prediction of Ago2-PB.(2) (C) Evolutionary distance tree of the selected species based on the NCBI taxonomy database; for comparison, the distance between flies and crustaceans (length of red line) is greater than the distance between primates and bony fish (length of blue lines). (D) Plot of the Jensen-Shannon distance to a background distribution based on a gapped multiple sequence alignment of the full-length Ago2 protein sequences from the species indicated above (absolute conservation = 1.0). The start of the folded region (sequence …PLPLPP… in the orange connector sequence in a) within the alignment is indicated. (E) The N-terminal IDR domains are highly basic (each data point represents one of the species). Top: comparison of the amino acid frequencies between the folded portions of Ago2 and the N-terminal domain (separated at the …PLPLPP… motif in the connector segment, note the different scales for the axes). The longest Ago2 isoform for each species is selected, the sequence is separated in the folded and N-terminal part, and the amino acid frequency within each region is independently calculated for each species. Middle: comparison of the pI between the folded portions of Ago2 and the N-terminal domain. The pI is calculated separately for the N-terminal and folded regions. Bottom: comparison of the length-normalized net charge at pH 7 between the folded portions of Ago2 and the N-terminal domain. (F) Representation of a section of the structure of heparin. Heparin is a highly sulfated glycosaminoglycan polymer of varying size (6 to 30 kDa with the majority between 17 – 19 kDa) and degree of sulfation.

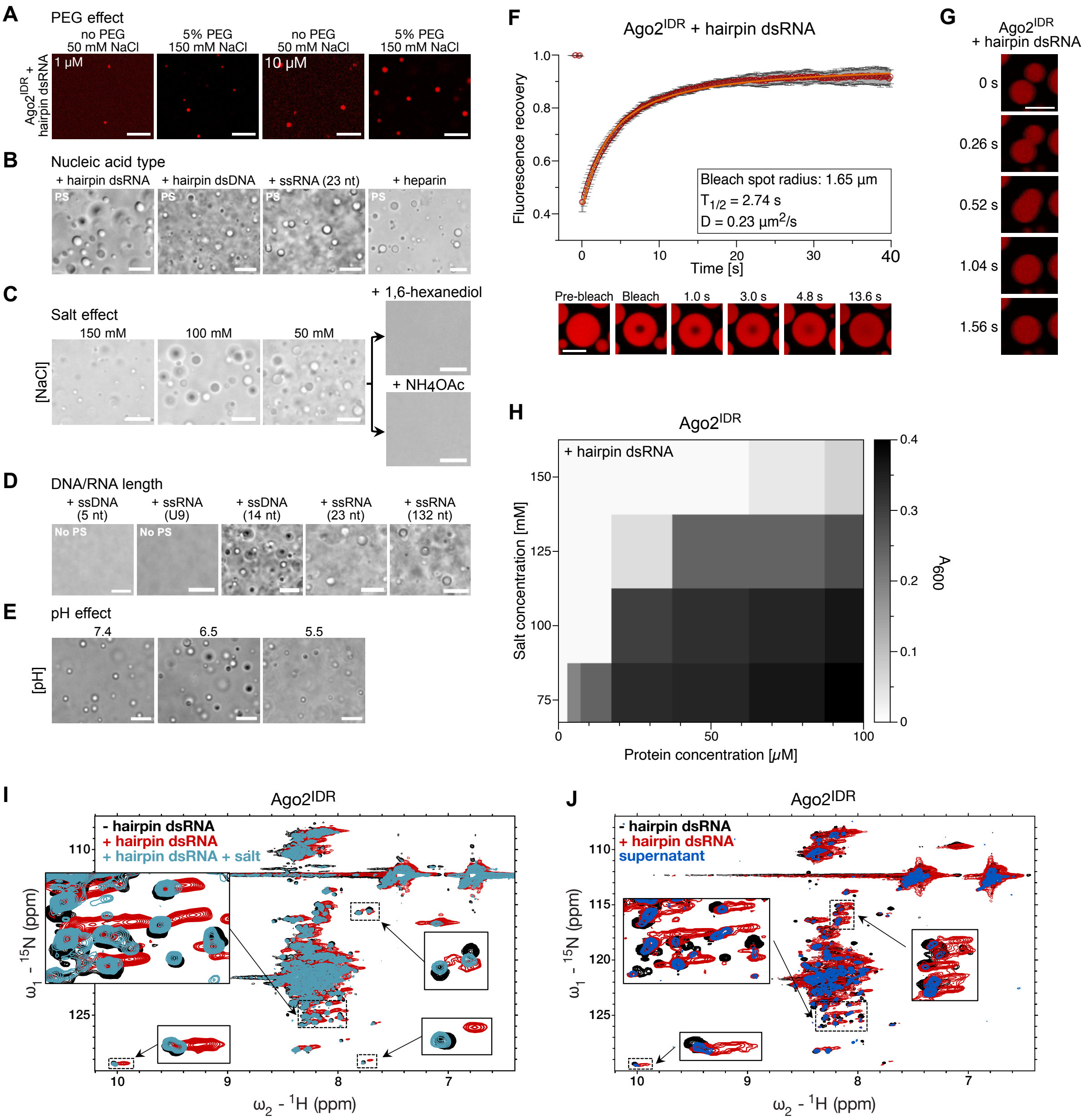

Figure S5. Nucleic acid-driven phase separation of Ago2^{IDR}. (A) Fluorescent microscopy images of Ago2^{IDR} in the presence of 21 bp hairpin dsRNA at low concentrations (1 and 10 µM Ago2^{IDR}, 25 µM RNA, PS-I buffer, or PS-II buffer with 5% PEG8000, scale bar 10 µm). (B) Effect of the nucleic acid type on phase separation. Nucleic acid-induced droplets of Ago2^{IDR} (50 µM, PS-I buffer) in the presence of 21 bp hairpin dsRNA, 21 bp hairpin dsDNA, 23 nt ssRNA (25 µM), and heparin (0.1 mg/ml) as a nucleic acid mimetic (Phase contrast microscopy, scale bar 10 µm). The corresponding oligonucleotides are shown in Table S1. (C) Salt-dependent phase separation of Ago2^{IDR}. Phase contrast microscopy images of phase separation of Ago2^{IDR} (50 µM, 20 mM sodium phosphate, pH 6.5) with 21 bp hairpin dsRNA (50 µM) at varying salt concentrations and after addition of 5% 1,6-hexanediol, 500 mM ammonium acetate. (D) Nucleic acid length-dependent droplet formation of Ago2^{IDR} (50 µM) in the presence of 200 ng/µL ssDNA (5 nt), ssRNA (U9), ssDNA (14 nt), ssRNA2 (23 nt) and ssRNA (134 nt) in PS-I buffer (Phase contrast microscopy). The corresponding oligonucleotides are shown in Table S1. (E) pH-dependent phase separation of Ago2^{IDR}. Ago2^{IDR} phase separation (25 µM, 50 mM NaCl) in the presence of 21 bp hairpin dsRNA (10 µM) at different pH (20 mM HEPES pH 7.4, 20 mM sodium phosphate pH 6.5 or 20 mM MES pH 5.5, phase contrast microscopy). (F) Fast fluorescence recovery of bleached spots in Ago2^{IDR}/21 bp hairpin dsRNA droplets in FRAP experiments confirm the liquid-like nature of the condensates (50 µM protein, PS-I buffer, bleach spot radius ≈ 1.65 µm, droplet radius ≈ 7.06 µm, scale

bar 10 μm). Top: fluorescence recovery curve over time. The error is calculated from 3 replicates of FRAP measurements of the same sample. Bottom: fluorescence microscopy images of one replicate at selected time points during the FRAP experiment. (G) Fusion of two $Ago2^{IDR}$/21 bp hairpin dsRNA droplets (50 μM protein, 25 μM RNA, PS-I buffer). (H) Turbidity assay (A_{600}) at increasing protein concentrations of $Ago2^{IDR}$ and salt concentrations (20 mM sodium phosphate, pH 6.5) in the presence of 21 bp hairpin dsRNA (25 μM). (I) NMR spectral changes of $Ago2^{IDR}$ upon phase separation (related to **Figure 3D**). Overlay of the ^{1}H–^{15}N HSQCs of $Ago2^{IDR}$ (100 μM, PS-I buffer, black), $Ago2^{IDR}$ with 21 bp hairpin dsRNA (100 μM, red) and after addition of 100 mM NaCl (light blue). (J) Overlay of the ^{1}H–^{15}N HSQCs of $Ago2^{IDR}$ (100 μM, PS-I buffer, black), $Ago2^{IDR}$ with 21 bp hairpin dsRNA (100 μM, red), and the supernatant after centrifugation of the NMR sample (blue) (related to **Figure 3D**).

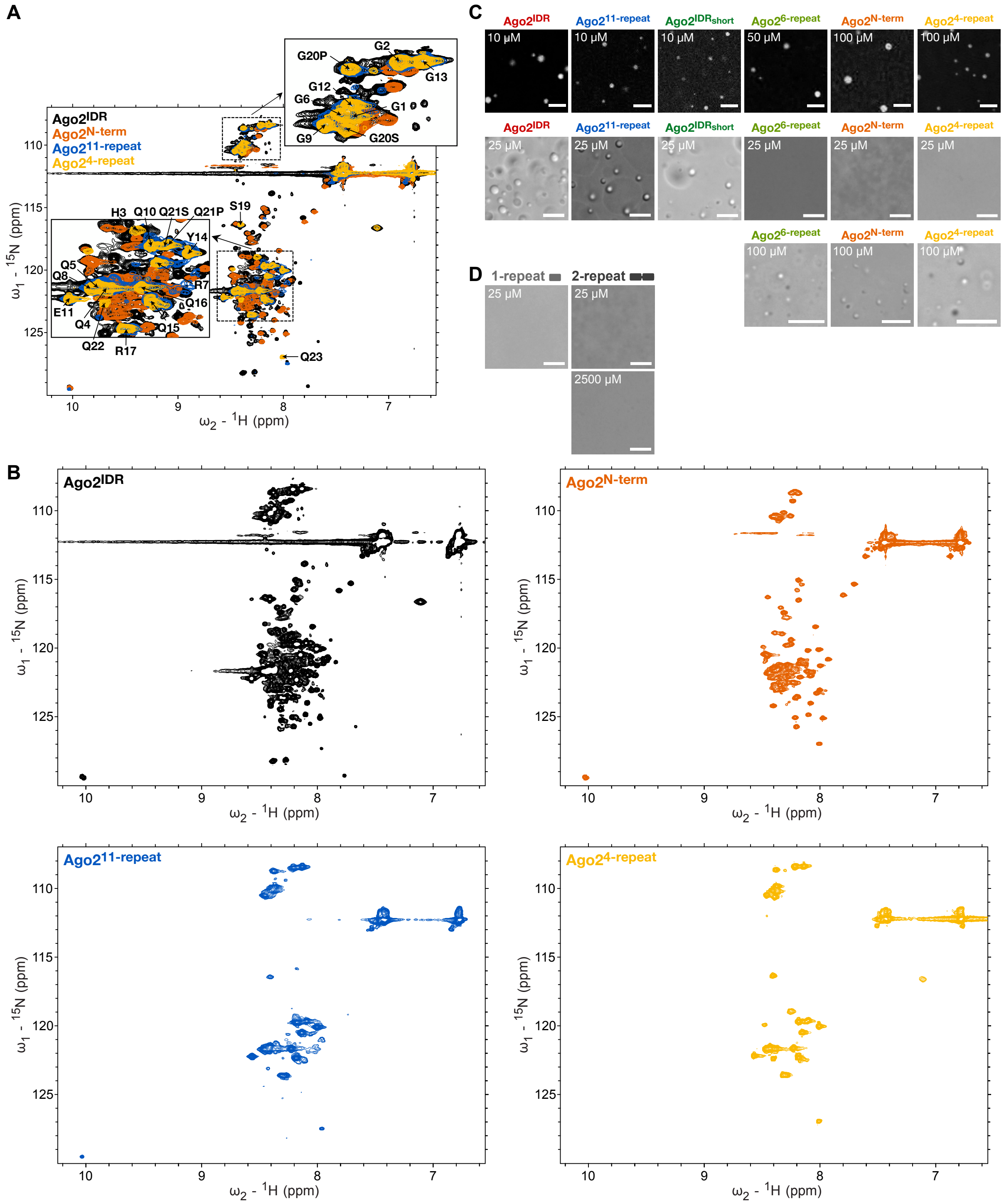

Figure S6. Phase separation of Ago2 constructs. (A) Overlay of the ^{1}H–^{15}N HSQC spectra of Ago2^{IDR} (black), Ago2$^{N\text{-}term}$ (orange), Ago2$^{11\text{-}repeat}$ (blue) and Ago2$^{4\text{-}repeat}$ (yellow) show good superimposition (PS-I buffer). The NMR assignments of Ago2$^{4\text{-}repeat}$ are shown in the zooms. (B) ^{1}H–^{15}N HSQC spectra of Ago2^{IDR} (black), Ago2$^{N\text{-}term}$ (orange), Ago2$^{11\text{-}repeat}$ (blue) and Ago2$^{4\text{-}repeat}$ (yellow) (C) Fluorescent and phase contrast microscopy images of phase separation of Ago2 sub-constructs (Ago2^{IDR}, Ago2$^{11\text{-}repeat}$, Ago2^{IDRshort} (10 μM and 25 μM), Ago2$^{6\text{-}repeat}$ (25, 50, 100 μM), Ago2$^{N\text{-}term}$ (25, 100 μM) and Ago2$^{4\text{-}repeat}$ (25, 100 μM)) in the presence of 21 bp hairpin dsRNA (25 μM) at room temperature (PS-I buffer, scale bar 10 μm). (D) Phase contrast microscopy images of Ago2$^{1\text{-}repeat}$ (25 μM) and Ago2$^{2\text{-}repeat}$ (25 μM, 2500 μM) in the presence of 21 bp hairpin dsRNA (25 μM) (PS-I buffer, scale bar 10 μm) show no phase separation.

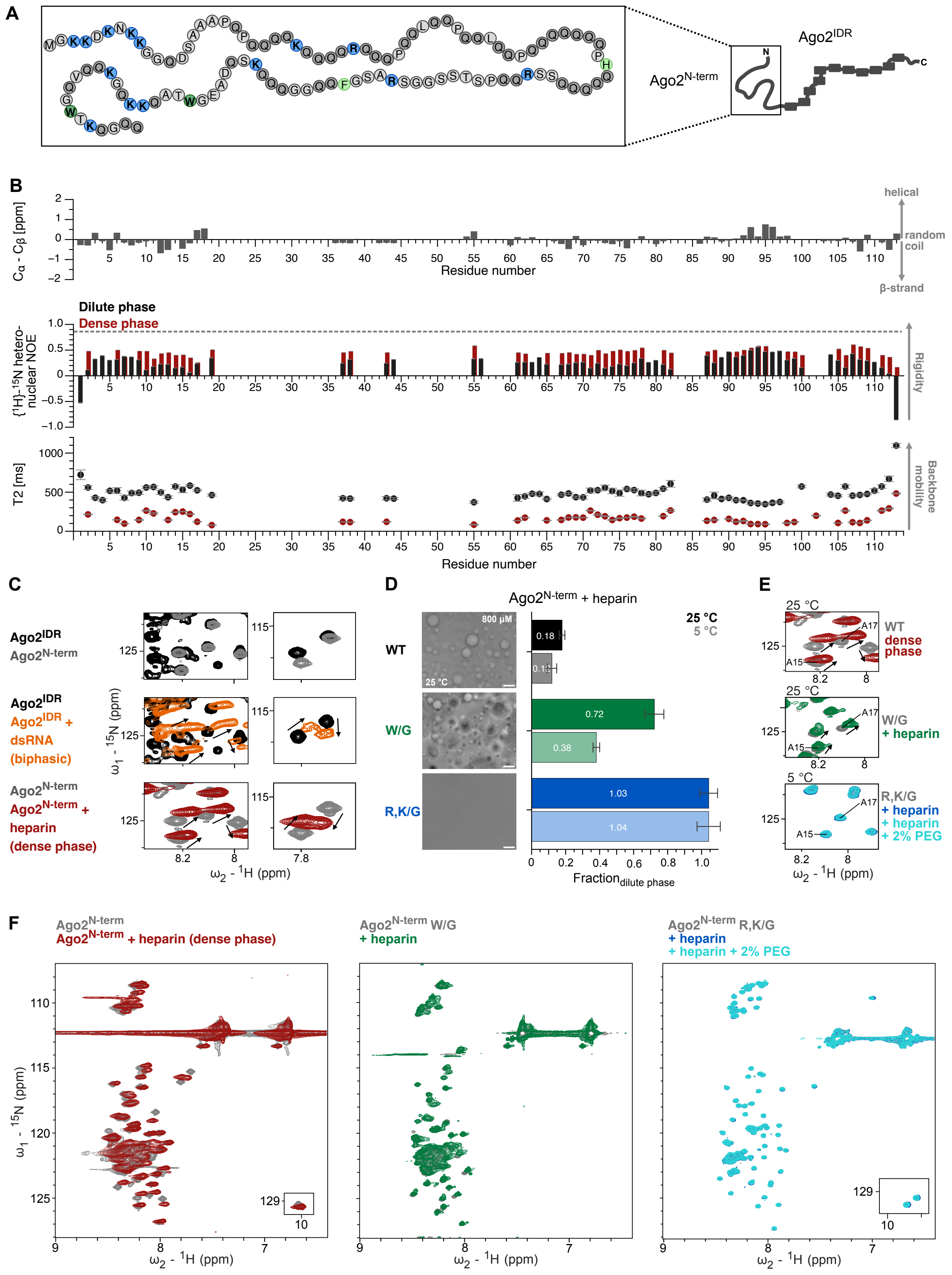

Figure S7. The dense phase of Ago2$^{N\text{-}term}$ shows characteristic NMR chemical shifts and phase separation is mediated by charged and aromatic sidechains. (A) Schematic representation of Ago2$^{N\text{-}term}$. Q, K/R, and aromatic residues are highlighted by dark grey, blue, and green-filled circles. (B) Top: secondary structure propensity by $^{13}C\alpha$ and $^{13}C\beta$ secondary chemical shifts confirm the unstructured nature of Ago2$^{N\text{-}term}$ (dilute phase). Bottom: comparison of the dilute and dense phase backbone dynamics by NMR ^{15}N relaxation experiments. ^{1}H-^{15}N heteronuclear nuclear Overhauser effect (NOE)

(error margins are estimated from spectral noise) and T_2 (error is obtained from fitting to the exponential function) of Ago2$^{N\text{-term}}$ (dilute phase, black) and condensed phase sample of Ago2$^{N\text{-term}}$ with heparin (dense phase, red). (C) ^{1}H–^{15}N HSQC spectral comparison of Ago2^{IDR} (black) and Ago2$^{N\text{-term}}$ before (gray, full ^{1}H–^{15}N HSQC spectra in **Supplementary Figure S6B**) and after phase separation induced by 21 bp hairpin dsRNA for Ago2^{IDR} (biphasic, orange, full ^{1}H–^{15}N HSQC spectra in **Supplementary Figure S5I**) and heparin for Ago2$^{N\text{-term}}$ (dense phase, red, full ^{1}H–^{15}N HSQC spectra in **Supplementary Figure S7F**). (D) Mutational effect on phase separation of Ago2$^{N\text{-term}}$ analyzed by phase contrast microscopy (800 μM protein, 2 mg/ml heparin, PS-I buffer, scale bar 10 μm) and sedimentation assay (800 μM protein, PS-I buffer) with heparin (2 mg/ml) at 25 °C (dark color) and 5 °C (light color). Error bars represent the standard deviation of three replicates from distinct samples. In Ago2$^{N\text{-term}}$ W/G (green) W93 and W107 are mutated to glycine, in Ago2$^{N\text{-term}}$ RK/G (blue) all arginines and lysines are mutated to glycines. (E) ^{1}H–^{15}N HSQC spectral comparison of Ago2$^{N\text{-term}}$ (dilute phase, gray) and with heparin (dense phase, red), Ago2$^{N\text{-term}}$ W/G (800 μM, PS-I buffer) without (gray) and with heparin (2 mg/ml, green) and Ago2$^{N\text{-term}}$ R,K/G (800 μM, PS-I buffer) without (gray) and with heparin (2 mg/ml, blue) and after addition of PEG8000 as a crowding reagent (2%, light blue). (F) Left: Overlay of the full ^{1}H–^{15}N HSQC spectra of Ago2$^{N\text{-term}}$ before (gray) and after phase separation induced by heparin (dense phase, red). Middle: Overlay of the full ^{1}H–^{15}N HSQC spectra of Ago2$^{N\text{-term}}$ W/G before (gray) and after addition of heparin (green). Right: Overlay of the full ^{1}H–^{15}N HSQC spectra of Ago2$^{N\text{-term}}$ R,K/G before (gray), after addition of heparin (blue) and after subsequent addition of 2% PEG (light blue). Related to **Supplementary Figure S7C** and **S7E**.

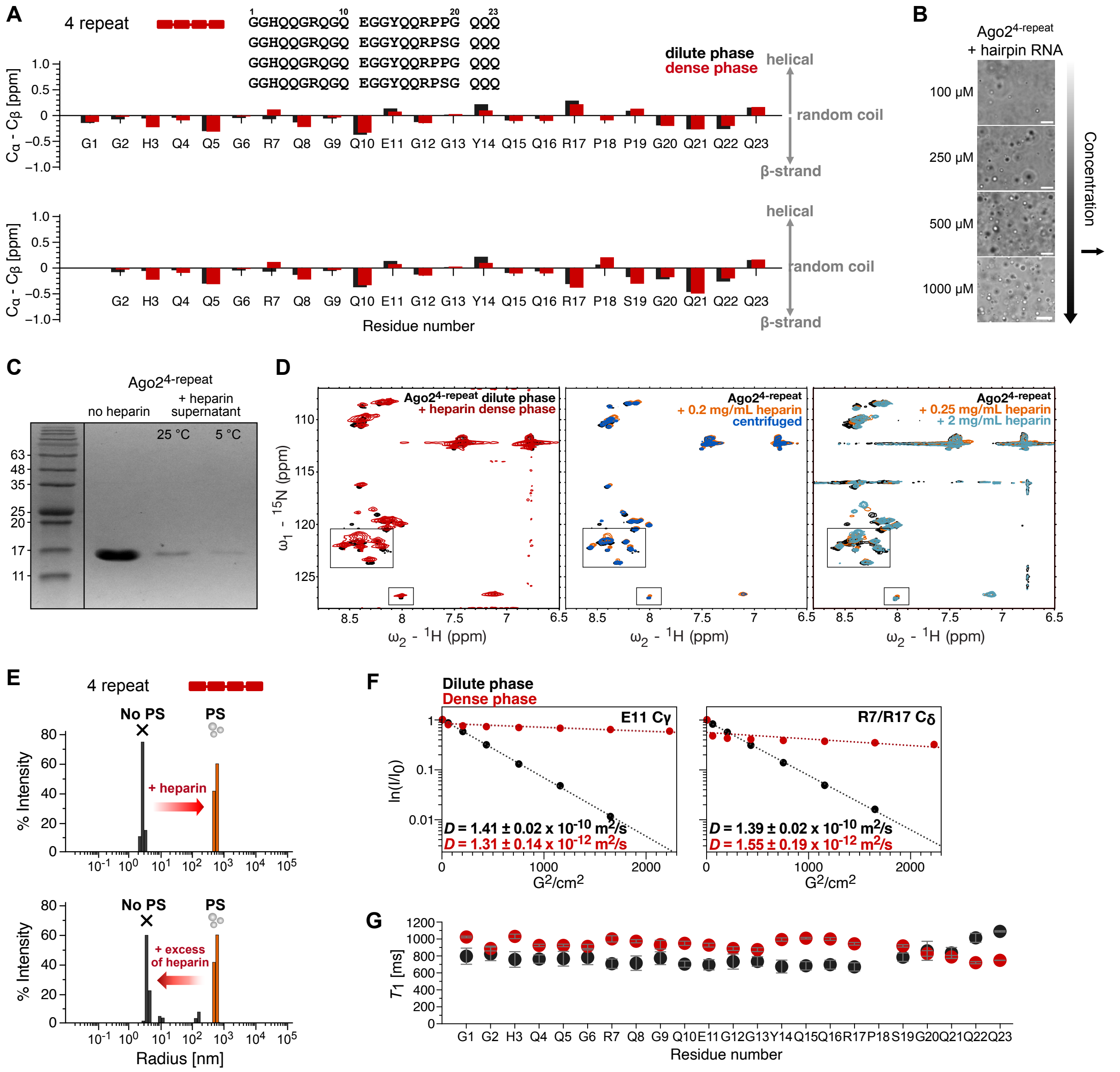

Figure S8. Determinants of Ago2$^{4\text{-repeat}}$ phase separation. (A) Comparison of secondary structure propensity by $^{13}C_{\alpha}$ and $^{13}C_{\beta}$ secondary chemical shifts of Ago2$^{4\text{-repeat}}$ dilute phase (black) and dense phase of Ago2$^{4\text{-repeat}}$ with heparin (red) show a similar pattern. (B) Concentration-dependent droplet formation of Ago2$^{4\text{-repeat}}$ in the presence of 21 bp hairpin dsRNA (25 µM, PS-I buffer, phase contrast microscopy). (C) SDS-Gel of Ago2$^{4\text{-repeat}}$ before adding heparin and of the supernatant after adding heparin and subsequent centrifugation at 25 °C and 5 °C (800 µM protein + 2 mg/ml heparin, samples are diluted 1: 20 for SDS-gel). (D) Comparison of the ^{1}H-^{15}N HSQC spectra of Ago2$^{4\text{-repeat}}$ samples. Left: Ago2$^{4\text{-repeat}}$ (100 µM, PS-I buffer, dilute phase, black) compared to the condensed phase sample of Ago2$^{4\text{-repeat}}$ with heparin (dense phase, red). Middle: Ago2$^{4\text{-repeat}}$ (dilute phase, black) compared to Ago2$^{4\text{-repeat}}$ (100 µM) with 0.2 mg/ml heparin to induce phase separation (biphasic, orange) and the supernatant after centrifugation of this sample (centrifuged, blue, PS-I buffer). Right: Ago2$^{4\text{-repeat}}$ (dilute phase, black) compared to Ago2$^{4\text{-repeat}}$ (100 µM) with 0.25 mg/ml heparin to induce phase separation (biphasic, orange) and after the addition of 2 mg/ml heparin (high heparin concentration, light blue, PS-I buffer) (related to **Figure 4B**, displayed parts are indicated with a box). (E) Reversible phase separation of Ago2$^{4\text{-repeat}}$ at high heparin concentrations. Top: DLS of Ago2$^{4\text{-repeat}}$ (100 µM, PS-I buffer) in the absence (black) and presence of heparin (0.2 mg/ml, orange). Bottom: DLS of Ago2$^{4\text{-repeat}}$ with heparin (orange) and after the addition of high concentrations of heparin (2 mg/ml, black). (F) Comparison of the diffusion coefficient from 2D ^{1}H-^{13}C diffusion experiments of Ago2$^{4\text{-repeat}}$ in the dilute phase and dense phase (related to **Figure 4C**). Normalized intensity ratio and fitted curves as a function of gradient

strength for $Ago2^{4\text{-repeat}}$ in the dilute phase (black, D ≈ 1.4 x 10^{-10} m^2/s) and dense phase (red, D ≈ 1.4 x 10^{-12} m^2/s) for residues E11 C_γ and R7/R17 C_δ. The error is estimated by the fit of the curves. (G) Comparison of the dilute and dense phase dynamics by NMR. T_1 (900 MHz ^1H Larmor frequency) of $Ago2^{4\text{-repeat}}$ (dilute phase, black 850 μM, PS-I buffer) and condensed phase sample of $Ago2^{4\text{-repeat}}$ with heparin (dense phase, red). The error is obtained from fitting to the exponential function. The different changes in ^{15}N T_1 for the last residues in the repeats in the dense and dilute phase indicate distinct dynamics of these regions.

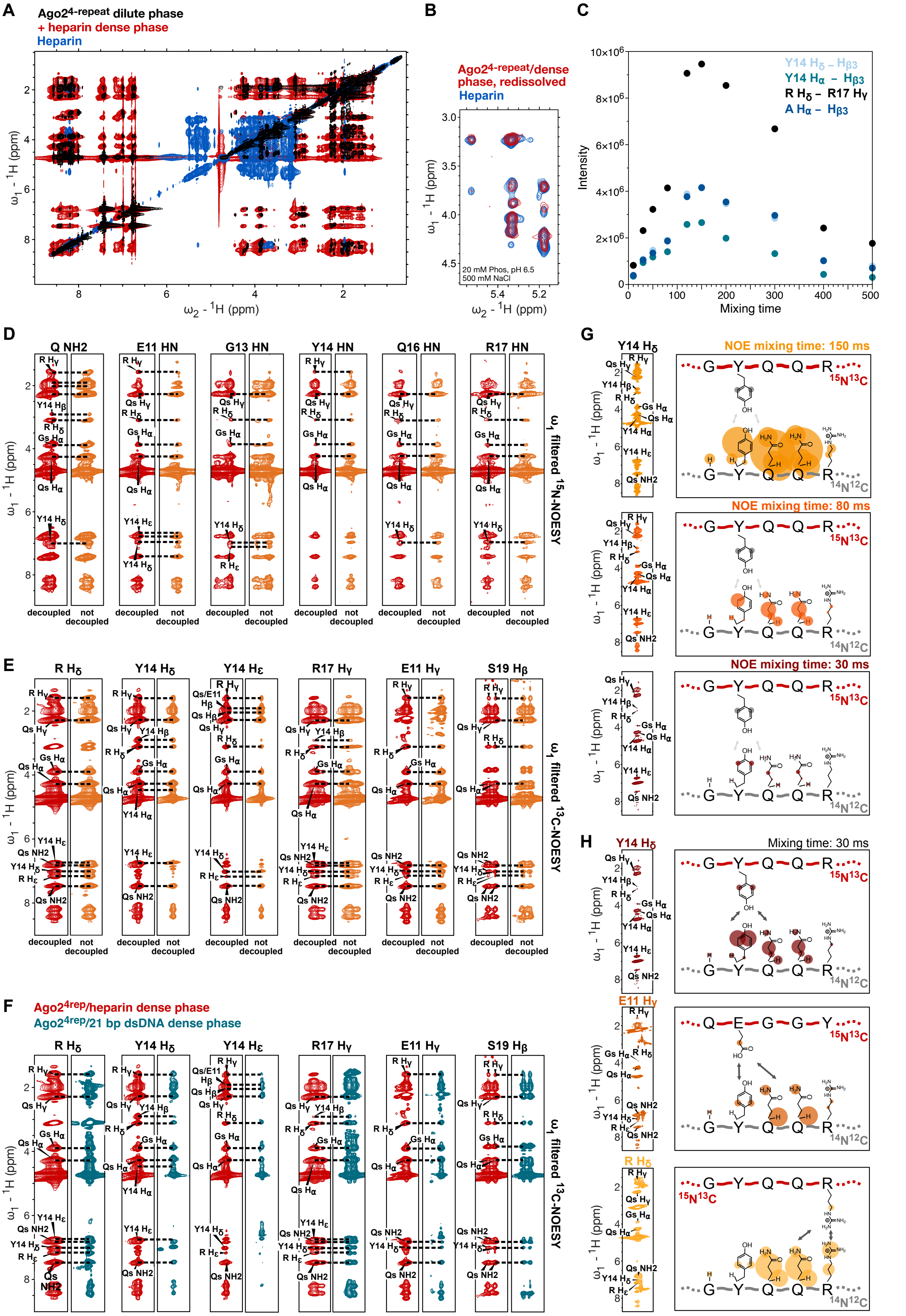

Figure S9. Analysis of interactions in the Ago2$^{4\text{-repeat}}$/heparin dense phase. (A) Overlay of the 2D-NOESY spectra (900 MHz ^{1}H Larmor frequency, 150 ms mixing time) of Ago2$^{4\text{-repeat}}$ (1.8 mM, PS-I buffer,

dilute phase, black), condensed phase sample of Ago2$^{4\text{-repeat}}$ with heparin (~ 20 mM, dense phase, red) and heparin (20 mg/ml, PS-I buffer, blue). (B) Heparin is present in the condensed phase. Section of the overlay of 2D ^{1}H,^{1}H NOESY (500 MHz ^{1}H Larmor frequency, 150 ms mixing time) of heparin (20 mg/ml, 20 mM Phos pH 6.5, 500 mM NaCl, blue) and the dense phase of Ago2$^{4\text{-repeat}}$ with heparin dissolved in 20 mM Phos pH 6.5, 500 mM NaCl to remove phase separation (red) (C) Intensity of selected NOE cross peaks of 2D NOESYs (1.2 GHz ^{1}H Larmor frequency) of Ago2$^{4\text{-repeat}}$/heparin dense phase at different mixing times (10 ms, 30 ms, 50 ms, 80 ms, 120 ms, 150 ms, 200 ms, 300 ms, 400 ms, and 500 ms). (D) Intermolecular contacts between H^N and H^N or H^C protons in the Ago2$^{4\text{-repeat}}$ dense phase. Strips of selected residues of the ω_1-filtered, ω_3-^{15}N-edited NOESY of Ago2$^{4\text{-repeat}}$ dense phase (1.2 GHz ^{1}H Larmor frequency, 150 ms NOE mixing time) with (red) and without (orange) ^{13}C decoupling. Comparison with the spectrum without ^{13}C decoupling (orange) ensures the observed NOEs are intermolecular. Intermolecular NOE cross peaks for which assignment was possible are labeled in black. (E) Intermolecular contacts of H^C protons in the Ago2$^{4\text{-repeat}}$ dense phase. Strips of selected residues of the ω_1-filtered, ω_3-^{13}C-edited NOESY-HSQC of Ago2$^{4\text{-repeat}}$ dense phase (1.2 GHz ^{1}H Larmor frequency, 150 ms NOE mixing time) with (red) and without (orange) ^{13}C and ^{15}N decoupling show intermolecular NOEs. Comparison with the spectrum without decoupling (orange) ensures the observed NOEs are intermolecular. Intermolecular NOE cross peaks for which assignment was possible are labeled in black. (F) Comparison of the intermolecular contacts of H^C protons in the Ago2$^{4\text{-repeat}}$/heparin dense phase and Ago2$^{4\text{-repeat}}$/21 bp dsDNA dense phase. Strips of selected residues of the ω_1-filtered, ω_3-^{13}C-edited NOESY-HSQC of Ago2$^{4\text{-repeat}}$/heparin dense phase (red) and Ago2$^{4\text{-repeat}}$/21 bp dsDNA dense phase (DNA concentration: ~ 35 mM, ocean). NOE mixing time for both spectra was 150 ms (1.2 GHz ^{1}H Larmor frequency). Intermolecular NOE cross peaks for which assignment was possible are labeled in black (G) Strips of tyrosine H_δ of the ω_1-filtered, ω_3-^{13}C-edited NOESY-HSQC (1.2 GHz ^{1}H Larmor frequency) of Ago2$^{4\text{-repeat}}$/heparin dense phase at NOE mixing times of 150 ms (yellow), 80 ms (orange), and 30 ms (brown). At each mixing time, the intermolecular contacts shown by NOE signals are exemplarily visualized by circles around the atom on a part of the protein sequence. The size of the circle reflects the intensity of the NOE signal (a decrease in mixing time leads to a decreased signal intensity). (H) Intermolecular contacts in the Ago2$^{4\text{-repeat}}$ dense phase. Strips of selected residues (dark red: Y14 H_δ, orange: E11 H_γ, yellow: R H_δ) of the ω_1-^{13}C, ^{15}N-filtered ω_3-^{13}C-edited NOESY-HSQC of Ago2$^{4\text{-repeat}}$ dense phase (30 ms NOE mixing time, 1.2 GHz ^{1}H Larmor frequency). Intermolecular NOE cross peaks for which assignment was possible are labeled in black. The intermolecular contacts shown by the NOE signals are exemplarily visualized by circles around the atoms on a part of the protein sequence. The size of the circle reflects the intensity of the NOE signal.

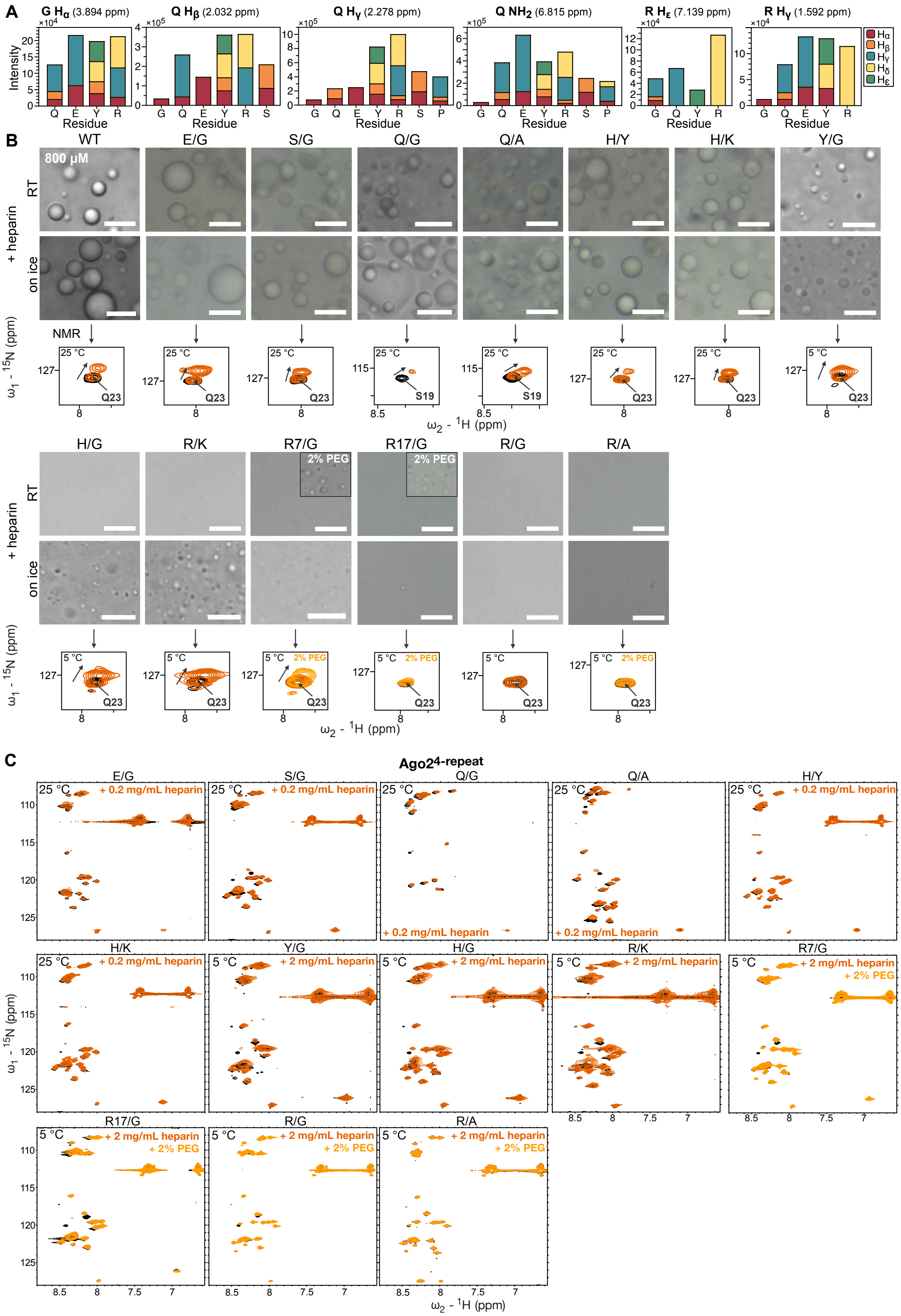

Figure S10. The effect of mutations in Ago2$^{4\text{-repeat}}$ on condensate formation. (A) Intensities of intermolecular NOEs (ω_1-^{13}C, ^{15}N-filtered ω_3-^{13}C-edited NOESY-HSQC, mixing time 80 ms) of

predominantly resolved ^1H positions to ^1H, ^{13}C strips (related to **Figure 5C**). Stacked bars represent different residue types. NOE intensities are corrected for the number of residues in the amino acid sequence. Only unambiguously assigned residues are reported. (B) Phase separation of Ago2$^{4\text{-repeat}}$ mutants is decreased compared to the WT. Top: phase contrast microscopy images of phase separation of Ago2$^{4\text{-repeat}}$ WT and mutants (800 μM, PS-I buffer) with heparin (2 mg/ml) at room temperature (RT) and on ice (scale bar 10 μm). Bottom: comparison of the overlay of ^1H–^{15}N HSQC spectra of Ago2$^{4\text{-repeat}}$ WT, Ago2$^{4\text{-repeat}}$ S/G, Ago2$^{4\text{-repeat}}$ Q/G, Ago2$^{4\text{-repeat}}$ Q/A, Ago2$^{4\text{-repeat}}$ H/K, Ago2$^{4\text{-repeat}}$ H/Y (100 μM, PS-I buffer, dilute phase, black) in the presence and absence of 0.2 mg/ml heparin at 25 °C and other mutants (Y/G, H/G, R/K, R7/G, R17G, R/G and R/A, 800 μM, PS-I buffer, dilute phase, black) in the presence and absence of 2 mg/ml heparin at 5 °C (orange). For Ago2$^{4\text{-repeat}}$ R7/G, R17G, R/G and R/A, the addition of 2% PEG8000 as a crowding reagent was used to induce phase separation (yellow). (C) Overlay of the full ^1H–^{15}N HSQC spectra of Ago2$^{4\text{-repeat}}$ S/G, Q/G, Q/A, H/K, H/Y (100 μM, PS-I buffer, dilute phase, black) at 25 °C with 0.2 mg/ml heparin and the other Ago2$^{4\text{-repeat}}$ mutants (Y/G, H/G, R/K, R7/G, R17G, R/G and R/A) at 5 °C (800 μM, PS-I buffer, dilute phase, black) with 2 mg/ml heparin (orange). For Ago2$^{4\text{-repeat}}$ R7/G, R17G, R/G and R/A 2% PEG8000 are added as a crowding reagent (yellow).

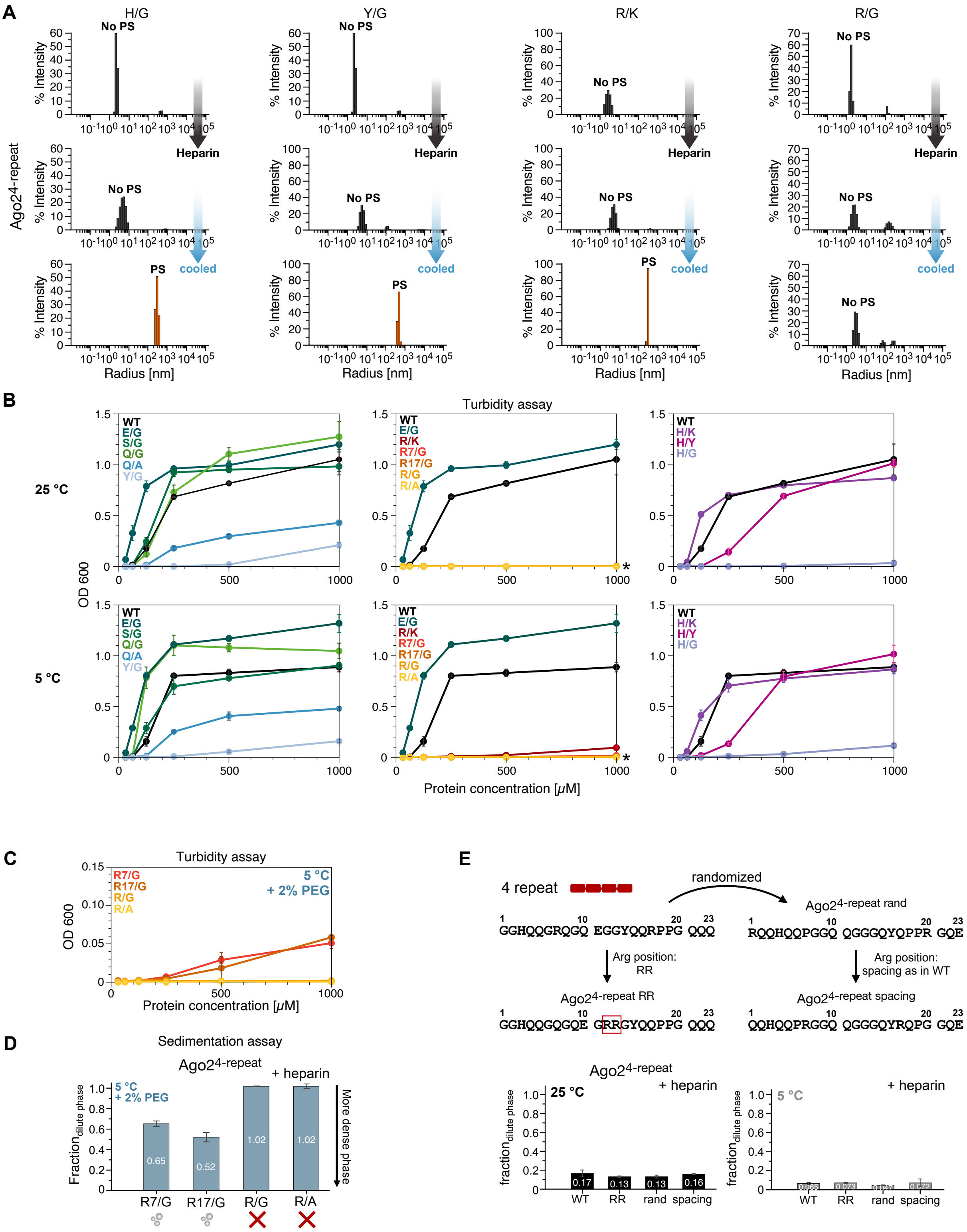

Figure S11. The effect of mutations in Ago2$^{4\text{-repeat}}$ on condensate formation. (A) Temperature-dependent particle size changes in DLS of some of the Ago2$^{4\text{-repeat}}$ mutants (Y/G, H/G, R/K and R/G). Top: dilute phase of Ago2$^{4\text{-repeat}}$ mutants (Y/G, H/G, R/K and R/G) in the absence of heparin (black, 100 μM). Middle: Ago2$^{4\text{-repeat}}$ mutants (Y/G, H/G, R/K and R/G) in the presence of heparin (0.2 mg/ml) at 25 °C. Bottom: Ago2$^{4\text{-repeat}}$ mutants (Y/G, H/G, R/K and R/G) with heparin (0.2 mg/ml) after cooling on ice (orange, PS-I buffer). (B) Turbidity assay (OD_{600}) at increasing concentrations of the different mutants of Ago2$^{4\text{-repeat}}$ (PS-I buffer) in the presence of heparin (0.5 mg/ml) at 25 °C (top) and at 5 °C (bottom). Left: Differential effect of various mutations on phase separation. Middle: Effect of the mutation of charged residues on phase separation (R/K, R7/G, R17/G, R/G, and R/A curves at 25 °C and R7/G, R17/G,

R/G and R/A curves at 5 °C overlap in the turbidity assay because they don't show phase separation at those conditions, marked with *). Right: Effect of the mutation of Histidine on phase separation. Error bars represent the standard deviation. (C) Turbidity assay (OD_{600}) with 2% PEG at increasing concentrations of the mutants of Ago2$^{4\text{-repeat}}$ that don't show phase separation in other conditions (R7/G, R17/G, R/G, R/A) in the presence of heparin (0.5 mg/ml) at 5 °C (PS-I buffer, 2% PEG). (D) Sedimentation assay of Ago2$^{4\text{-repeat}}$ mutants that don't show phase separation in other conditions (R7/G, R17/G, R/G, R/A) (800 μM, PS-I buffer) with heparin (2 mg/ml) and PEG (2%) at 5 °C. Error bars represent the standard deviation of three replicates from distinct samples. (E) Effect of amino position on condensate formation. Top: schematic representation of the mutations introduced in Ago2$^{4\text{-repeat}}$ to test the effect of amino acid position on condensate formation. In Ago2$^{4\text{-repeat RR}}$, the two arginines in each repeat are positioned consecutively. In Ago2$^{4\text{-repeat rand}}$, the amino acid sequence of one repeat is randomized and the mutant consists of four 23 amino acid repeats with this randomized sequence. In Ago2$^{4\text{-repeat spacing}}$, the arginines in the sequence of Ago2$^{4\text{-repeat rand}}$, are positioned in the same spacing as in the WT sequence. Bottom: quantitative comparison of protein concentration in the dilute phase after inducing phase separation of Ago2$^{4\text{-repeat}}$ WT and mutants (Ago2$^{4\text{-repeat RR}}$, Ago2$^{4\text{-repeat rand}}$ and Ago2$^{4\text{-repeat spacing}}$, 800 μM, PS-I buffer) with heparin (2 mg/ml) at 25 °C (black) and 5 °C (grey).

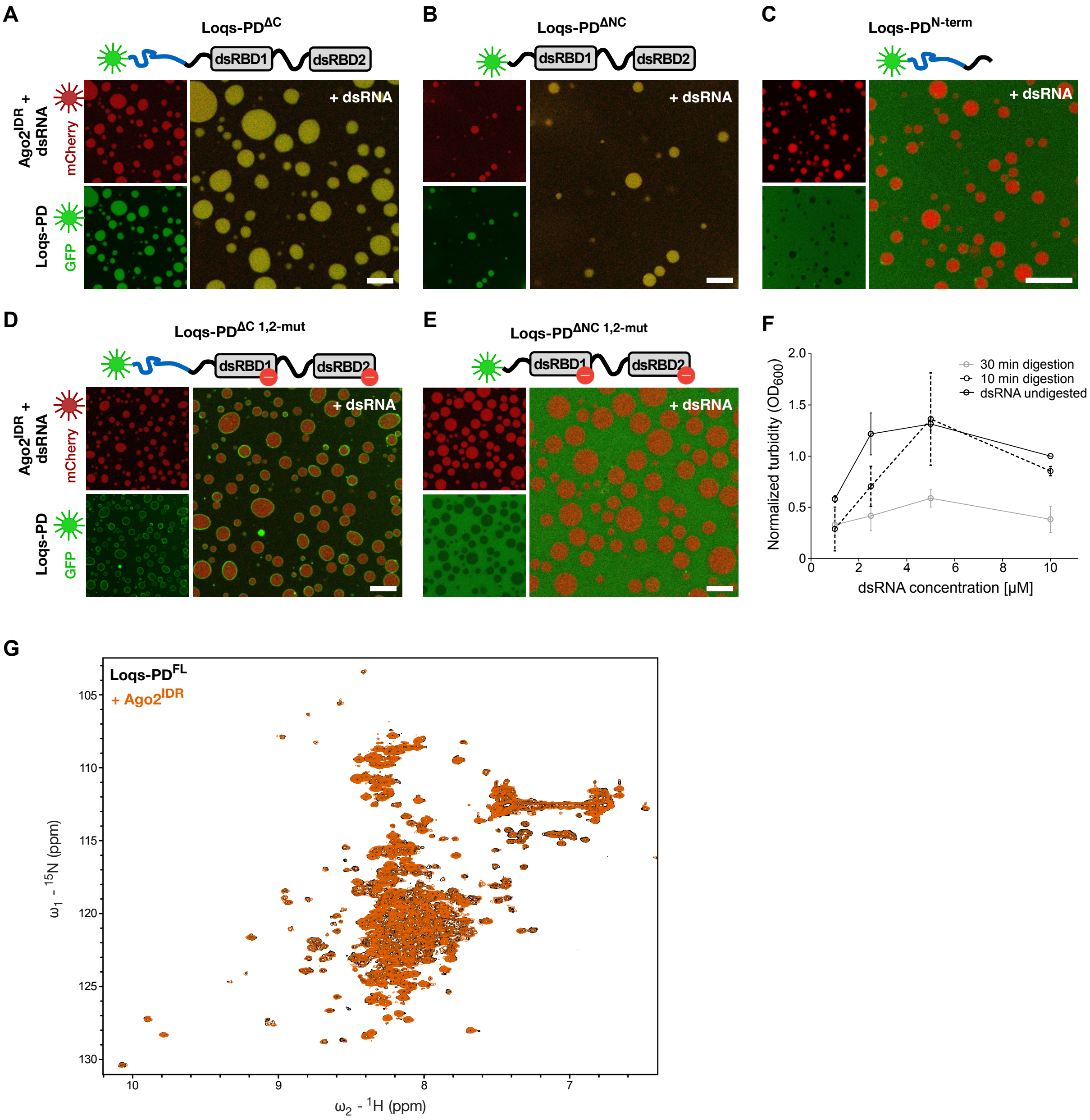

Figure S12. Coacervation of Ago2^{IDR}, dsRNA, and Loqs-PD mutants. (A) Coacervation of Ago2^{IDR} in the presence of 21 bp hairpin dsRNA with Loqs-PD$^{\Delta C}$ (C-terminal deletion mutant) by fluorescent microscopy (50 µM protein, 25 µM RNA, PS-I buffer, scale bar 10 µm). (B) Coacervation of Ago2^{IDR} in the presence of 21 bp hairpin dsRNA with Loqs-PD$^{\Delta NC}$ (N- and C-terminal deletion mutant) by fluorescent microscopy (50 µM protein, 25 µM RNA, PS-I buffer, scale bar 10 µm). (C) Coacervation of Ago2^{IDR} in the presence of 21 bp hairpin dsRNA with Loqs-PD$^{N\text{-}term}$ (only N-terminus) by fluorescent microscopy (50 µM protein, 25 µM RNA, PS-I buffer, scale bar 10 µm). (D) Coacervation of Ago2^{IDR} in the presence of 21 bp hairpin dsRNA with Loqs-PD$^{\Delta C\ 1,2\text{-}mut}$ (mutant with C-terminal region deleted and KK to AA mutations in dsRBD1 and dsRBD2 that hinder RNA binding) by fluorescent microscopy (50 µM protein, 25 µM RNA, PS-I buffer, scale bar 10 µm). (E) Coacervation of Ago2^{IDR} in the presence of 21 bp hairpin dsRNA with Loqs-PD$^{\Delta NC\ 1,2\text{-}mut}$ (mutant with N- and C-terminal region deleted and KK to AA mutations in dsRBD1 and dsRBD2 that hinder RNA binding) by fluorescent microscopy (50 µM protein, 25 µM RNA, PS-I buffer, scale bar 10 µm). (F) Pre-digestion of dsRNA into shorter products reduces its potential to induce phase separation; Ago2^{IDR} was 20 µM in all cases, the dsRNA concentration was titrated and indicated corresponding to the original length of 160 nt. Three independent experiments were performed, and the turbidity was normalized to the value measured for the highest

concentration of undigested RNA on each measurement day (average +/- SD plotted). (G) No direct interaction of Loqs-PD and Ago2^{IDR}. NMR titration of Loqs-PD^{FL} (black, 100 μM, PS-II buffer, 292 K) with Ago2^{IDR} (orange, 50 μM).

SUPPLEMENTARY TABLES

Table S1. Oligonucleotide constructs used in this study.

Name	Source	Purpose	Sequence
21bp hairpin dsRNA	this study	NMR, phase separation assay	GGAGCUUUCAAAAUGAUCUCCGUGAG GAGAUCAUUUUGAAAGCUCC
Template_hairpin RNA	this study	DNA template for *in vitro* transcription	GGAGCTTTCAAAATGATCTCCTCACGGA GATCATTTTGAAAGCTCCTATAGTGAGT CGTATTA
21bp hairpin dsDNA	IDT	NMR, phase separation assay	GGAGCTTTCAAAATGATCTCCGTGAGG AGATCATTTTGAAAGCTCCTATAGTGAG TCGTATTA
ssRNA (23 nt)	Tants et al.(3)	Phase separation assay	UUCCUCGAAAGUUUUACUAGAGG
FITC labeled bantam dsRNA	Tants et al.(3)	Phase separation assay	Sense: UGAGAUCAUUUUGAAAGCUGAU*U antisense: UCAGCUUUCAAAAUGAUCUCACU
ssDNA (5 nt)	Eurofins	Phase separation assay	TTTGT
ssRNA (U9)	Dharmacon	Phase separation assay	UUUUUUUUU
ssDNA (14 nt)	Eurofins	Phase separation assay	TTTGTAAAATTTTG
ssRNA (134 nt)	Ebersberger et al.(4)	Phase separation assay	GGUAUCGUUUGAAAACUUUCCAAUUU CUUUCUCUUCUCGCCCCCCUCACUUU UCCUACCUUUACUCUUCUUUUCCAUU UUCUUUCUUCUUUGCUGUUCUCUCU CUUUUCCAUGAAUACCAUUUUUUCCC UGCAG
Heparin sodium salt from porcine intestinal mucosa	Sigma-Aldrich (CAS: 9041-08-1)	NMR, phase separation assay	

Table S2. Cytoplasmic Loqs-PD concentrations of four replicates (related to Fig. S1J).

Replicate	$I_{cells/purified}$	$c_{cytoplasmic}$ Loqs-PD [μM]
1	1.8	20
2	0.6	6.6
3	0.6	8.8
4	0.8	6.5

Table S3. Protein constructs used in this study.

Insert	Purpose	Vector backbone	Amino acid boundaries	Mutations
Loqs-PD^{FL}	NMR, phase separation assay	pET24	1-359	
GFP-Loqs-PD^{FL}	Phase separation assay	pETM11	1-359	
GFP-Loqs-PD$^{FL\ 1,2\text{-}mut}$	Phase separation assay	pETM11	1-359	K189A, K190A, K301A, K302A
Loqs-PD$^{N\text{-}term}$	NMR, phase separation assay	pET24	1-129	
GFP-Loqs-PD$^{N\text{-}term}$	Phase separation assay	pETM11	1-129	
Loqs-PD$^{\Delta N}$	NMR, phase separation assay	pET24	129-359	
GFP-Loqs-PD$^{\Delta N}$	Phase separation assay	pETM11	129-359	
GFP-Loqs-PD$^{\Delta N\ 1,2\text{-}mut}$	Phase separation assay	pETM11	129-359	K189A, K190A, K301A, K302A
Loqs-PD$^{\Delta C}$	NMR, phase separation assay	pET24	1-322	
Loqs-PD$^{\Delta C\ 1\text{-}mut}$	NMR, phase separation assay	pET24	1-322	K189A, K190A
Loqs-PD$^{\Delta C\ 2\text{-}mut}$	NMR, phase separation assay	pET24	1-322	K301A, K302A
Loqs-PD$^{\Delta C\ 1,2\text{-}mut}$	NMR, phase separation assay	pET24	1-322	K189A, K190A, K301A, K302A
GFP- Loqs-PD$^{\Delta C}$	Phase separation assay	pETM11	1-322	
GFP- Loqs-PD$^{\Delta C\ 1,2\text{-}mut}$	Phase separation assay	pETM11	1-322	K189A, K190A, K301A, K302A
Loqs-PD$^{\Delta NC}$	NMR, phase separation assay	pET24	129-322	
Loqs-PD$^{\Delta NC\ 1\text{-}mut}$	NMR, phase separation assay	pET24	129-322	K189A, K190A
Loqs-PD$^{\Delta NC\ 2\text{-}mut}$	NMR, phase separation assay	pET24	129-322	K301A, K302A
GFP-Loqs-PD$^{\Delta NC}$	Phase separation assay	pETM11	129-322	
GFP-Loqs-PD$^{\Delta NC\ 1,2\text{-}mut}$	Phase separation assay	pETM11	129-322	K189A, K190A, K301A, K302A
Myc-Loqs-PD(5)	Microscopy in cells	pCaSpeR-derivative	1-359	
Myc-R2D2(6)	Microscopy in cells	pCaSpeR-derivative	FL	

GFP-Loqs-PD^{WT}	Fluorescence microscopy in cells	pCaSpeR-derivative	1-359	
GFP-Loqs-PD$^{1,2\text{-}mut}$	Fluorescence microscopy in cells	pCaSpeR-derivative	1-359	K189A, K190A, K301A, K302A
GFP-Loqs-PD$^{1\text{-}mut}$	Fluorescence microscopy in cells	pCaSpeR-derivative	1-359	K189A, K190A
GFP-Loqs-PD$^{2\text{-}mut}$	Fluorescence microscopy in cells	pCaSpeR-derivative	1-359	K301A, K302A
IDR-mCherry-Ago2	Fluorescence microscopy in cells	pCaSpeR-derivative	1-386-mCherry-387-1208	mCherry tag is localized between Ago2 IDR and N-terminal domain due to cleavage if tagged N-terminally
GFP-Ago2	Fluorescence microscopy in cells	pCaSpeR-derivative	1-386-muGFP-387-1208	Monomeric ultrastable [mu]GFP tag is localized between Ago2 IDR and N-terminal domain due to cleavage if tagged N-terminally
GFP-Ago2$^{\Delta IDR}$	Fluorescence microscopy in cells	pCaSpeR-derivative	muGFP-387-1208	ΔIDR, aa 1-386 missing
IDR-GFP	Fluorescence microscopy in cells	pCaSpeR-derivative	1-386-muGFP-STOP	Truncated protein with a stop codon after the IDR + muGFP
GFP	Fluorescence microscopy in cells	pCaSpeR-derivative	muGFP	muGFP protein (control)
Ago2$^{N\text{-}term}$	NMR, phase separation assay	pET24	1-113	
Ago2$^{N\text{-}term\ W/G}$	NMR, phase separation assay	pET24	1-113	W93G, W107G
Ago2$^{N\text{-}term\ R,K/G}$	NMR, phase separation assay	pET24	1-113	All Rs and Ks to G
Ago2$^{1\text{-}repeat}$	NMR, phase separation assay	pET24	114-136	
Ago2$^{2\text{-}repeat}$	NMR, phase separation assay	pET24	137-159	
Ago2$^{4\text{-}repeat}$	NMR, phase separation assay	pET24	137-182	
Ago2$^{4\text{-}repeat}$ S/G	NMR, phase separation assay	pET24	137-228	All Ss to Gs

Ago2$^{4\text{-repeat}}$ E/G	NMR, phase separation assay	pET24	137-228	All Es to Gs
Ago2$^{4\text{-repeat}}$ Q/G	NMR, phase separation assay	pET24	137-228	All Qs to Gs
Ago2$^{4\text{-repeat}}$ Q/A	NMR, phase separation assay	pET24	137-228	All Qs to As
Ago2$^{4\text{-repeat}}$ H/Y	NMR, phase separation assay	pET24	137-228	All Hs to Ys
Ago2$^{4\text{-repeat}}$ H/K	NMR, phase separation assay	pET24	137-228	All Hs to Ks
Ago2$^{4\text{-repeat}}$ H/G	NMR, phase separation assay	pET24	137-228	All Hs to Gs
Ago2$^{4\text{-repeat}}$ Y/G	NMR, phase separation assay	pET24	137-228	All Ys to Gs
Ago2$^{4\text{-repeat}}$ 2R/K	NMR, phase separation assay	pET24	137-228	All Rs to K
Ago2$^{4\text{-repeat}}$ R7/G	NMR, phase separation assay	pET24	137-228	R7 in each repeat to G
Ago2$^{4\text{-repeat}}$ R17/G	NMR, phase separation assay	pET24	137-228	R17 in each repeat to G
Ago2$^{4\text{-repeat}}$ R/G	NMR, phase separation assay	pET24	137-228	All Rs to Gs
Ago2$^{4\text{-repeat}}$ R/A	NMR, phase separation assay	pET24	137-228	All Rs to As
Ago2$^{4\text{-repeat RR}}$	NMR, phase separation assay	pET24	137-228	Rs in each repeat spaced next to each other
Ago2$^{4\text{-repeat rand}}$	NMR, phase separation assay	pET24	137-228	Repeat sequence randomized
Ago2$^{4\text{-repeat spacing}}$	NMR, phase separation assay	pET24	137-228	Repeat sequence randomized and Rs in same spacing as in WT
Ago2$^{6\text{-repeat}}$	NMR, phase separation assay	pET24	106-252	
Ago2^{IDRshort}	NMR, phase separation assay	pET24	1-113, 4rep, 366-413	
Ago2$^{11\text{-repeat}}$	NMR, phase separation assay	pET24	106-370	
Ago2^{IDR}	NMR, phase separation assay	pET24	1-413	
mCherry-Ago2^{IDR}	Phase separation assay	pET24	1-413	

Table S4. Buffers used for phase separation assays and NMR experiments in this study.

Buffer name	Buffer composition
PS-I buffer	20 mM Phos, pH 6.5, 50 mM NaCl
PS-II buffer	20 mM Phos, pH 6.5, 150 mM NaCl

SUPPLEMENTARY REFERENCES

1. Schmidts, I., Böttcher, R., Mirkovic-Hösle, M. and Förstemann, K. (2016) Homology directed repair is unaffected by the absence of siRNAs in Drosophila melanogaster. *Nucleic Acids Research*, **44**, 8261-8271.
2. Lancaster, A.K., Nutter-Upham, A., Lindquist, S. and King, O.D. (2014) PLAAC: a web and command-line application to identify proteins with prion-like amino acid composition. *Bioinformatics*, **30**, 2501-2502.
3. Tants, J.N., Fesser, S., Kern, T., Stehle, R., Geerlof, A., Wunderlich, C., Juen, M., Hartlmüller, C., Böttcher, R., Kunzelmann, S. *et al.* (2017) Molecular basis for asymmetry sensing of siRNAs by the Drosophila Loqs-PD/Dcr-2 complex in RNA interference. *Nucleic Acids Res*, **45**, 12536-12550.
4. Ebersberger, S., Hipp, C., Mulorz, M.M., Buchbender, A., Hubrich, D., Kang, H.-S., Martínez-Lumbreras, S., Kristofori, P., Sutandy, F.X.R., Llacsahuanga Allcca, L. *et al.* (2023) FUBP1 is a general splicing factor facilitating 3' splice site recognition and splicing of long introns. *Molecular Cell*, **83**, 2653-2672.e2615.
5. Hartig, J.V., Esslinger, S., Böttcher, R., Saito, K. and Förstemann, K. (2009) Endo-siRNAs depend on a new isoform of loquacious and target artificially introduced, high-copy sequences. *Embo j*, **28**, 2932-2944.
6. Hartig, J.V. and Förstemann, K. (2011) Loqs-PD and R2D2 define independent pathways for RISC generation in Drosophila. *Nucleic Acids Res*, **39**, 3836-3851.
